# Effectiveness of Psychosocial Interventions Targeting Hazardous and Harmful Alcohol Use and Alcohol-Related Symptoms in Low- and Middle-Income Countries: A Systematic Review

**DOI:** 10.3389/fpsyt.2020.00768

**Published:** 2020-08-07

**Authors:** Melissa Preusse, Frank Neuner, Verena Ertl

**Affiliations:** ^1^ Clinical Psychology and Psychotherapy, Department of Psychology, Bielefeld University, Bielefeld, Germany; ^2^ vivo international, Konstanz, Germany; ^3^ Department of Clinical Psychology and Biopsychology, Catholic University Eichstätt-Ingolstadt, Eichstätt, Germany

**Keywords:** alcohol, addiction, intervention, treatment, low- and middle-income country, systematic review

## Abstract

**Background:**

In low- and middle-income countries (LMIC), the mismatch between the number of individuals needing and those receiving treatment for alcohol use disorders (AUD) is substantial. In order to provide suggestions for the scaling up of effective service provision we systematically reviewed the current evidence on the effectiveness of AUD-focused psychosocial interventions in LMIC.

**Methods:**

We used a systematic review methodology following the PRISMA guidelines. Twelve electronic databases listing published and grey literature were searched and only randomized-controlled trials (RCTs) were included. Where possible, effect sizes were calculated using Hedges' *g* indices.

**Results:**

Twenty-one RCTs conducted in 15 different LMIC between 1992 and 2018 fulfilled inclusion criteria. Most studies employed brief one-on-one interventions facilitated by trained primary care staff. Eighty-six percent of RCTs based their interventions on the principles of motivational interviewing (MI) with the majority supplementing MI-based interventions with alcohol-tailored elements of cognitive-behavioral therapy (CBT). The remaining RCTs employed CBT-components exclusively. Just over 40% of studies included in quantitative analyses (n=17) yielded an at least medium-sized effect (*g*≥.50) of the respective intervention compared to alcohol-related and unrelated control conditions or waiting list. Only half of the trials implementing the widely applied MI-based approaches (or MI-based approaches blended with CBT-elements) were superior to their respective control conditions.

**Conclusion:**

To date, a relatively small number of RCTs investigating AUD-focused treatments has been conducted in LMIC. The majority of between condition effect size estimates were small and no type of intervention can clearly be recommended over another. No RCTs were conducted in conflict-affected areas in LMIC although they would merit particular attention since AUD is often linked to trauma-related mental health disorders. More RCTs in LMIC are required and alternatives to MI-based approaches should be investigated. This systematic review summarizes properties of effective interventions and provides implications for future research.

## Introduction

Excessive alcohol use and the resulting consequences pose a major challenge to health systems globally, and particularly to those of low- and middle-income countries [LMIC; defined according to World Bank country classification (1)]. The most recent World Health Organization (WHO) status report on alcohol and health (2) found that, although most alcohol per capita is consumed in wealthier countries, the alcohol-attributable burden of disease (3) per unit of consumed alcohol is higher in LMIC [e.g. (4, 5)]. A higher burden of disease implies higher morbidity and mortality risks due to diseases for which alcohol has a detrimental effect, including cardiovascular diseases, cirrhosis of the liver, neuropsychiatric disorders, and unintentional injuries. In developing settings poor living conditions and limited access to health care can perpetuate and exacerbate such medical conditions and thus further increase the burden of disease (3, 6). Other factors strongly associated with a higher burden of disease include risky and potentially harmful patterns of drinking (e.g. drinking to intoxication, drinking in public places) which were found to be more prevalent in LMIC compared to upper middle- and high-income countries with comparable levels of consumption (7, 8). Furthermore, unrecorded alcohol consumption (i.e., home-made alcohol, illegal alcohol products, or alcohol not officially made for human consumption) is widespread in LMIC, and Rehm and colleagues (9) presume detrimental health consequences beyond the impact of ethanol alone.

Although individuals with severe alcohol-related problems or alcohol dependence are at the highest risk of experiencing such alcohol-attributable harm, the burden on health care and social systems resulting from non-dependent, but *harmful or hazardous use*, is assumed to be even greater with the majority of alcohol-related problems attributable to this group of drinkers. This is not surprising since harmful or hazardous drinkers simply constitute the much larger group within a population [e.g. (10)]. The WHO defines *hazardous* drinking as “pattern of alcohol consumption that increases the risk of harmful consequences for the user or others,” and *harmful* alcohol use as “alcohol consumption that results in consequences to physical and mental health” (11).

Epidemiological research from LMIC has revealed very high prevalence rates of hazardous, harmful, or dependent drinking, reaching up to 28% in Tanzania (12), 22% in India (13), 40% in Namibia (14), 31% in Ethiopia (15), and 21% in Uganda (16). Studies predominantly used male, random or convenience samples from the general population. Special concern has been raised about alcohol consumption among LMIC-populations affected by conflict and/ or forced displacement (17) where even higher rates of risky drinking have been found, though the evidence base is still generally weak [for reviews see: (18–20)]. More recent epidemiological studies investigating prevalences of hazardous drinking among refugees, internally displaced persons (IDP) and former IDPs found high rates of 23% in Nepal (21), 28% in Georgia (22), and 32% (23), respectively 46% (24) in Northern Uganda.

While the alcohol-attributable health and economic burden placed upon affected populations is significant, the strains related to social harm are just as immense. From an economic perspective, impaired health is resulting in the loss of productivity of affected individuals thereby contributing to the persistence of poverty of whole regions (3, 25, 26). Furthermore, financial problems due to alcohol consumption and stigmatization are adverse effects not limited to the individual drinker, but directly affecting his or her family as well. Often, families of alcohol abusers are unable to pay for their children’s education, experience undernutrition, lack other essential needs, and experience marginalization and isolation (27–30).

Beyond impacting the family’s material needs, alcohol abuse has detrimental effects on the mental health of the drinkers themselves and of the individuals living with them. Proximal effects of drinking, such as impaired cognitive functioning and emotional lability, act as potential facilitators of tension and conflict within families (31, 32). Moreover, research including some studies from LMIC, frequently suggests associations between alcohol abuse and comorbid psychopathology such as depression, anxiety disorders, suicidal ideation, and posttraumatic stress disorder [PTSD; (5, 33–35)]. Various pathways have been hypothesized to explain this relationship linking hazardous drinking to the development, maintenance and exacerbation of psychological problems [e.g. (36)]. Moreover, studies have shown high levels of psychological distress among family members of alcohol abusers as well as dysfunctional family dynamics including multiple forms of violent behavior. In several LMIC-based studies, alcohol-related symptoms are among the most consistently found risk factors for intimate partner violence [IPV; e.g. (37–40)] and violence against children (41). In ongoing and post-conflict areas the relationship between excessive drinking and domestic violence seems to be magnified (42), which is particularly detrimental for LMIC-settings where the majority of the world’s most violent crises are currently taking place (43). This relationship has emerged even in studies controlling for traumatic experiences and psychopathologies in male respondents [in Sri Lanka (44) and Uganda (45, 46)].

High prevalence rates and severe negative consequences indicate the urgent need for alcohol-focused interventions in LMIC-settings as well as their scientific evaluation. With the launch of the Mental Health Gap Action Programme [mhGAP; (47)] in 2008 the WHO drew attention to the substantial mismatch between the number of people needing treatment and those receiving treatment for alcohol use disorders (AUD) in low-resource contexts. In fact, among all mental disorders, globally, the treatment gap for AUD was found to be the widest with nearly 80% of affected individuals remaining untreated (47), most of them residing in LMIC. This finding was replicated in a more recent cross-sectional study conducted in four LMIC where the population-level treatment gap was estimated to be between 94.9% and 97.2% for AUD (48). The research base on the efficacy of alcohol treatments delivered in high income countries (HIC) is quite substantial with numerous studies evaluating screening and brief intervention (SBI) programs; a concept involving systematic screening procedures plus structured interventions of short duration [1–4 sessions (11)]. Counseling approaches based on motivational enhancement, in particular, have been extensively examined and are recommended as first-line interventions by WHO’s mhGAP for implementation in routine health-care settings, though evidence on their effectiveness is mixed [for reviews see e.g. (49, 50)]. The most commonly used rationale is the principle of motivational interviewing (MI) which was first proposed by Miller in 1983 and then further elaborated by Miller and Rollnick in 1991 and constitutes a “guiding style for enhancing intrinsic motivation to change” (51). In further research, a number of elements were found to be recurring features in effective brief interventions. These features, represented by the acronym FRAMES: Feedback, Responsibility, Advice, Menu, Empathic, and Self-efficacy (52, 53), have subsequently been used by the WHO-affiliated brief alcohol interventions and are still referenced by brief interventions today. Other common interventions applied in HIC-trials include simple structured advice, cognitive behavioral therapy, or giving out leaflets on alcohol consumption [e.g. (54)].

Despite the evident global health significance of the issue, there is a lack of comprehensive review-level data regarding the effectiveness of AUD-focused interventions in LMIC. There are plenty of reviews condensing findings from high-income countries [e.g. (55–59)] with few including some data from trials based in developing and transitional countries [e.g. (60–65)]. However, as alcohol researchers have emphasized [e.g. (66, 67)], findings derived from HIC may not be generalizable to LMIC settings due to a number of reasons, including context-specific health issues, structural differences regarding the availability of resources, variations in drinking patterns and types of alcohol, and the severity of alcohol-related symptoms and consequences. A small number of reviews focused exclusively on alcohol treatments in LMIC, however, these do not include effect size calculations or do not describe systematic literature searches (66, 68). Furthermore, such reviews are divergent in scope as they focus on LMIC within specific geographical regions such as Sub-Sahara Africa (69, 70) or solely on middle-income countries (71). Others address wider spectrums of mental (72) or substance use disorders in general (73, 74) with alcohol being only one aspect. To our knowledge, reviews on alcohol intervention trials based in conflict-torn populations are, to date, nonexistent with only a few authors trying to raise awareness on the topic [e.g. (18, 24, 75, 76)]

With this review we aim to give an updated comprehensive overview of the currently implemented psychosocial interventions, its components, and their current state of evidence to serve as an orientation for practitioners as well as for future intervention trials. Therefore, the objective of the present study is to systematically review treatment trials aiming to reduce hazardous and harmful drinking and alcohol-related symptoms in LMIC and to summarize the evidence regarding their effectiveness.

## Methods

This systematic review was conducted in accordance with the Preferred Reporting Items for Systematic Reviews and Meta-Analyses (PRISMA) statement (77) [see **Supplementary Figure 1** for the PRISMA checklist]. We did not preregister this systematic review, however, we had a predefined review question, search strategy, inclusion and exclusion criteria, and risk of bias assessment strategy following the Cochrane guidelines for systematic reviews of interventions (78).

### Search Strategy and Inclusion/Exclusion Criteria

The search for eligible intervention studies was undertaken in the electronic databases PsychINFO, PubMed, PSYNDEX, Web of Science, Google Scholar, Cochrane Library (CENTRAL) using combinations of the following search terms: alcohol OR drink* AND treatment OR intervention OR program OR therapy AND “low- and middle-income countr*” OR “low-income countr*” OR “middle-income countr*” OR “developing countr*” OR “post-conflict” OR war OR “post-war”. In order to avoid the potential for publication bias an explicit search for grey literature (i.e., unpublished reports, master and dissertation theses) was conducted in the following databases using the same search terms described above: DissOnline, DART-Europe, EthOS, OATD, ProQuest, and Open Grey. Furthermore, prospective trial registration databases (Clinicaltrials.gov, ISRCTN Registry, INEBRIA, WHOLIS, PACTR) were searched for intervention research projects that fit the purpose of the review in terms of intervention focus and outcomes in order to overcome time-lag bias. Authors of such projects were contacted requesting any conference contributions, unpublished reports or manuscripts in preparation or under review they would be willing to provide. There were no limitations regarding the publication dates of the studies. The search was conducted up to August 5^th^, 2019.

**Figure 1:**
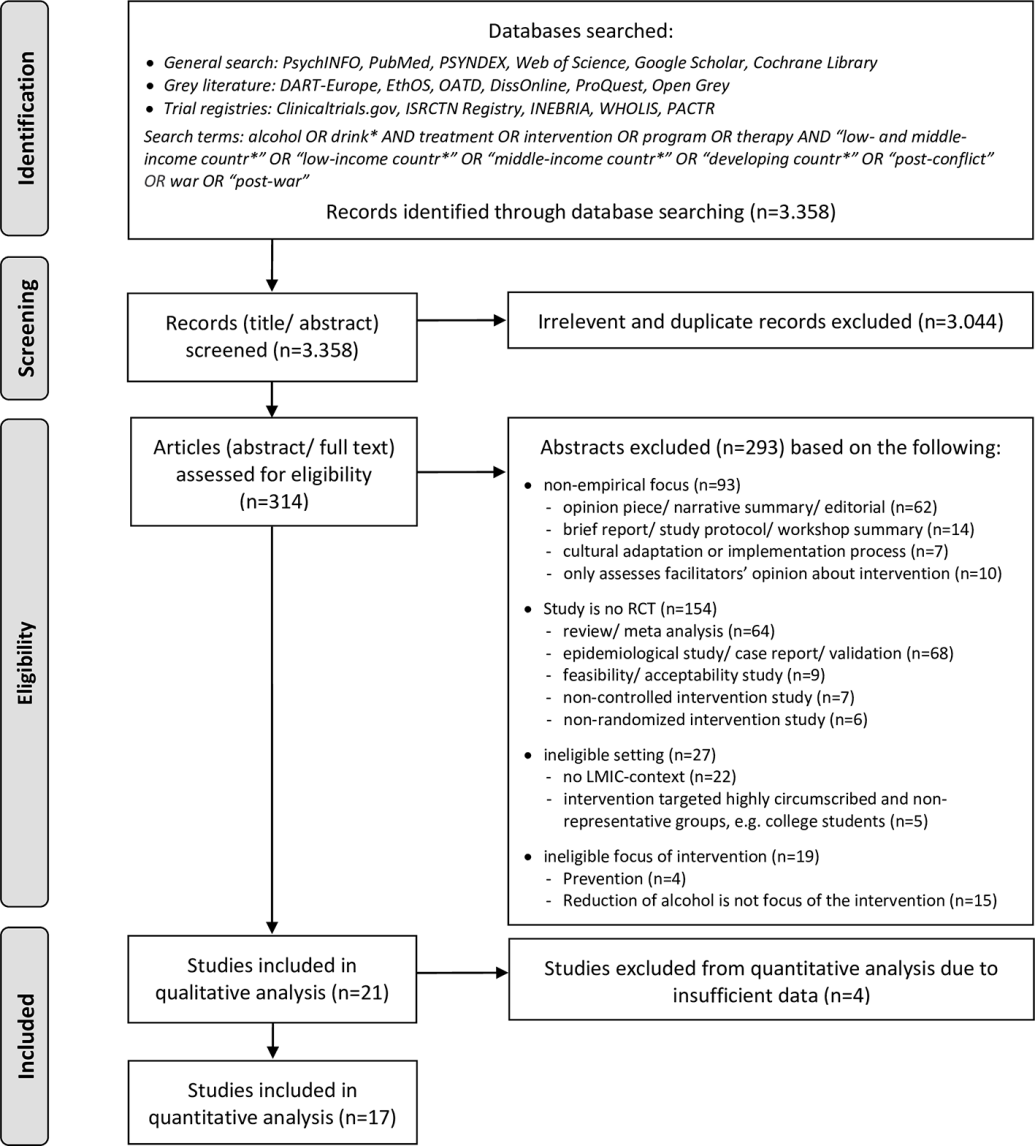
PRISMA flow diagram.

The inclusion criteria were oriented along the PICOS (populations, interventions, comparators, outcomes, study designs) elements. Regarding the populations (P), the studies had to address individuals identified as hazardous or harmful drinkers through systematic clinical screening procedures. Due to the fact that many studies defined lower but not upper limits for their screening instruments (even if explicitly aiming at hazardous or harmful and not dependent drinkers) we were unable to exclude studies whose samples included dependent drinkers. Therefore, we decided during the process that determining dependent drinking as an excluding factor was not practicable, although we had previously considered this restriction. The presence of comorbid disorders (including abuse of other substances apart from alcohol) was not an exclusion criterion. Our aim was to identify intervention studies that recruited participants in primary health care, social care, or community-based settings in LMIC. We excluded treatment trials which targeted highly circumscribed subgroups that did not represent general LMIC populations, mainly particularly privileged groups such as university students. In terms of interventions (I), we applied the following inclusion criteria: psychosocial intervention specifically aiming to reduce consumption of alcohol, implemented in a low- or middle-income country [according to World Bank country classification (1)]. Interventions applied within other health programs (i.e., reproductive health programs, HIV-programs) were not excluded from the present review as long as the intervention focus included the reduction of alcohol use and respective outcomes were assessed. The simultaneous treatment of other drugs was also not an exclusion criterion as long as participants were also screened and treated for hazardous alcohol use. We excluded intervention trials targeting the “alcogenic environment” instead of individuals. For example, programs controlling availability or promotion of alcohol or trials evaluating the establishment of new policies were excluded from the present review. There were no restrictions regarding the type of comparison conditions (C). In terms of outcomes (O), eligible studies had to include at least some quantity- or frequency measure of alcohol consumption or a standardized scale assessing alcohol-related symptoms. Regarding study designs (S), we only included randomized-controlled trials, including pilot- and cluster-RCTs as the objective of the current review was to identify the best clinical evidence available for outpatient alcohol intervention components in LMIC. A total of 3.358 articles were identified from electronic database searching. As a first step, titles and abstracts were screened to remove irrelevant reports as well as duplicates, leading to 314 eligible abstracts. In a second step, these were analyzed against *a priori* set inclusion and exclusion criteria. In the event the information in the abstract was insufficient to determine inclusion or exclusion, the full text was retrieved and scanned. The final determination of whether a study met the eligibility criteria was made by two authors (MP, VE). Disagreements about whether a study was to be included were resolved by discussion. After analysis of the abstracts and/ or full texts, 293 studies were excluded. Most of them were non-empirical reports, non-RCTs or were excluded because of their non-LMIC setting (see **Figure 1** for details). The search resulted in a total number of 21 intervention studies. One study was a multicenter trial which applied the same research design and intervention in 10 different countries of which six were LMIC (79). We refer to it as *one* study throughout the text unless stated otherwise. An overview of the selection process is presented in **Figure 1**.

### Data Extraction and Analysis

Data was extracted on selected study-level characteristics for each primary study. Investigators were contacted to request data not available within the publications. Due to the heterogeneity of outcome assessments used across the studies and due to the large disparity regarding settings of recruitment, severity of alcohol-related symptoms, intervention intensities, and facilitators’ backgrounds, skill levels and trainings, combining outcomes into pooled effect sizes or other meta-analytic measures was not feasible. Instead, for studies which allowed us to do so, we calculated effect sizes based upon the most frequently applied outcome variables. These were grouped into two main categories: (1) *alcohol-related symptoms* using standardized assessment scales [i.e. Alcohol Use Disorder Identification Test, AUDIT (80) or Alcohol, Smoking and Substance Involvement Screening Test, ASSIST (81)] and (2) *alcohol* *amount consumed per defined unit* (i.e., grams of ethanol consumed within a set time frame, typically per day or per drinking day). In the event a study reported more alcohol-related outcomes beyond these categories, preference was given to the dependent variable belonging to (1) or (2) in order to increase uniformity. When a study reported outcomes from both main categories we calculated effect sizes for both of them. We used the continuous outcome that was most similar to one of the two main categories (e.g. number of days or times alcohol was drunk within a certain time frame) only for studies that did not report outcomes categorizable into any of the two categories and where no relevant data was available after contacting the authors and grouped them under (3) *other outcomes*. To further improve comparability between effect size values we only included outcomes assessed on either one or both of the most frequently used follow-up (FU) time points, which were 3 and 6 months post-enrollment or -intervention. Consequently, shorter or longer assessment points (4–8 weeks, available in three studies; 12 months, available in three studies) were not considered for quantitative analyses.

Whenever possible, we calculated two types of effect size values for each study: *within-condition effect sizes* comparing pre- and posttreatment means in the intervention groups to assess main effects of intervention over time, and *between-condition effect sizes* comparing intervention and comparison groups on group x time interaction effects. We further segregated effect sizes according to comparison condition. Comparison conditions were categorized into control groups that did not focus on alcohol use in any way (alcohol-*un*related control groups; e.g. interview assessments only, nutrition intervention, wellness promotion, routine medical care) and those that did provide some information or advice concerning drinking (alcohol-related control groups; e.g. psycho-education on hazardous alcohol use, education leaflets informing about responsible drinking, simple advice to cut down on drinking, personalized feedback on AUDIT-score). For studies which employed different experimental add-on conditions (e.g. MI-based intervention in one group and MI-based intervention complemented by cognitive-behavioral techniques in the other group), we included both conditions in the effect size calculations.

According to Lakens’ (82), effect sizes for correlated or dependent measurements (within-condition effect sizes) and independent measurements (between-condition effect sizes) should be computed as Hedges' *g* indices. Lakens (82) recommends the use of Hedge’s *g* over Cohen’s *d* as the Bessel-correction makes it a less biased measure in the estimation of the population variance. Following Lakens suggestion we used Hedge’s *g_av_* for within-treatment-condition and Hedge’s *g_s_* for between-condition effect sizes. Hedge’s *g_av_* uses the average standard deviation as a standardizer while Hedge’s *g_s_* uses the pooled standard deviation. All effect sizes were calculated using Lakens’ (83) calculation sheet for effect sizes. For the interpretation of Hedge’s *g* coefficients the commonly used benchmarks (84) of small (d ≥ .20), medium (d ≥ .50), and large (d ≥ .80) as well as very large [d ≥ 1.30; (85)] effect sizes were applied. Where numeric information required for the calculation of effect sizes was insufficient or not available in the published article or by contacting the authors (e.g. missing standard deviations) we excluded the study from the quantitative, but not qualitative analysis. Regarding missing participant data, we employed the type of data that was available in the published articles for calculation of effect sizes. If both intention-to-treat and complete case data were available preference was given to intention-to-treat data.

### Assessment of Methodological Quality

Assessment of risk of bias was guided by the Cochrane Collaboration’s Risk of Bias Tool [CCRBT; (86)]. The tool suggests different domains of potential bias: selection bias (adequacy of randomization and allocation concealment), performance bias (blinding of participants), detection bias (blinding of outcome assessment), attrition bias (amount, nature or handling of incomplete outcome data), and reporting bias (due to selective outcome reporting). The assessment involves categorizing studies as having a low, high, or unclear risk of bias in these areas using the set of criteria listed within the tool. As blinding of intervention facilitators is virtually impracticable in trials involving behavioral interventions, within the category of performance bias we only assessed risk of bias regarding the extent to which subjects knew about their allocation and the potential bias associated with this knowledge. For studies where treatment manuals were used intervention procedures described in studies were cross-checked for adherence to the procedures described in the respective manuals.

## Results

### Study Characteristics

Twenty-one studies met our inclusion criteria. Years of publication ranged between 1992 (79) and 2018 (87) with 18 articles published in or after 2010.

#### Countries and Settings 

The interventions were implemented in 15 different LMIC. Although the trial settings were quite diverse, the majority of alcohol interventions were integrated into routine primary care services or were conducted within established health promotion or disease prevention programs at community health centers.

#### Participants 

Across all studies, a total of 6.488 participants were randomized to the trial conditions with total sample sizes per study ranging from 75 to 1,196 participants. Out of the studies that reported their participants’ mean age the youngest sample was 21.8 years old [SD=2.6; (88)] and the oldest sample’s average age was 42.0 years [SD=11.4; (89)]. The subjects of two trials (10%) were males only, three trials (15%) recruited only females, and the remaining 16 trials (76%) included subjects of both sexes.

Apart from the wide range of general inclusion criteria there was also substantial variation across the studies regarding their alcohol-related eligibility criteria. The majority of studies used interviewer-administered standardized self-report instruments, mostly AUDIT and ASSIST, however, studies were not consistent with regard to applied cut-off-scores. The diversity of alcohol-related inclusion criteria accounted for a rather heterogeneous overall sample in terms of symptom severity. The samples of six studies either completely (90, 91) or partially [40% (88), 63% (92), 73.2% (93), and “about 50%” (94)] consisted of alcohol-dependent individuals. In eight studies, dependent drinking is neither controlled for nor explicitly addressed, although the respective samples are likely to include dependent drinkers as the studies either used lower but no upper screening limits (95–99), did not specify a cut-off score at all (87), or used an upper score potentially including dependent drinkers (100). The remaining seven studies employed procedures attempting to rule out the presence of alcohol-dependence among participants by either adhering to the upper cut-off scores proposed in the screening tool manuals (89, 101–104) or determining clinical judgment of alcohol dependence as an excluding factor (79, 105). An overview of countries, settings and sample characteristics is given in **Table 1** as well as in **Supplementary Table 1**.

**Table 1:** Study characteristics.

Study	Country	Setting of recruitment	Sample (Number of S. randomized to conditions)	Individual or group format	Intervention facilitation and training^a^	Contents of intervention and control group(s) (main components of the alcohol-focused interventions in bold)		Duration per component/ session	No. of sessions
Assanangkorn-chai et al. (101)	Thailand	Eight hospitals and health centers in Southern Thailand	n=747, 2% female, age range: 16–65, 61% aged 16–25	Individual	BI: by co-author who had been trained in the technique Simple Advice (SA) by a research assistant	TG	**MI** *The ASSIST-linked brief intervention for hazardous and harmful substance use: manual for use in primary care* (81)	M=8.8 min, range 5–13 min (max. 15 min)	Single session
						CG	Simple advice: P. received feedback on their ASSIST score and its meaning, they were simply advised to stop or reduce substance use	M=3.9 min, range 3–6 min	Single session
Babor et al. (79)	6 LMICs: Bulgaria, Costa Rica, Kenya, Mexico, Former Sovjet Union, Zimbabwe	Combination of hospital settings, primary care clinics, work-sites and educational institutions	n=818 Bulgaria: n=98, 12.2% female, age: M=37.8, SD=n/r Costa Rica: n=36, female: n/r, age: n/r Kenya: n=203, 0% female, age: M=34.6, SD=n/r Mexico: n=196, 0% female, age: n/r Former Sovjet Union: n=156, 0% female, age: M=38.0, SD=n/r Zimbabwe: n=129, 7.8% female, age: n/r	Individual	“Health advisors” hired for the study who received 10–20 h of training by the principle investigators according to written guidelines (standardized across centers)	TG1	**Mainly behavioral techniques** *Problem-Solving-Manual*; strategies derived from the behavioral change model (106), consistent with principles of social learning theory and motivational psychology	40 min	Single session
						TG2	Simple advice on reduction of alcohol consumption	25 min	Single session
						CG	20 min health interview (WHO composite interview)	20 min	Single session
Kalichman et al. (95)	South Africa	Urban STI-clinic in Cape Town	n=143 (15% female, age: M=28.75, SD=5.6)	Individual	2 local bachelor degree-level counselors with minimal counseling experience outside the study protocol	TG	**MI + Intervention on HIV** Brief alcohol counseling model of the WHO tailored to the individual level of drinking (107) + HIV education component: facts about HIV-transmission and risk behaviors (not alcohol-related)	60 min	Single session
						CG	HIV education component (same as TG)	20 min	Single session
Kalichman et al. (96)	South Africa	Informal alcohol serving establishments (“shebeens”) in a suburban township in Cape Town	n=353 (67% female, age: M=34.1, SD=10.5)	Group (8–10 same gender P. facilitated by 2 counselors)	2 local bachelor degree-level counselors with minimal counseling experience outside the study protocol who received a 3-week training by project managers	TG	**MI + Intervention on HIV** Brief alcohol counseling model of the WHO tailored to the individual level of drinking (107) + HIV education component: facts about HIV-transmission and risk behaviors (not alcohol-related)	180 min	Single session
						CG	HIV education component (same as TG)	60 min	Single session
L’Engle et al. (102)	Kenya	HIV prevention centers in Mombasa offering services for female sex workers	n=818 (all female, age: M=27.5, SD=6.6, range 18–54)	Individual	Nurse counselors who received training in motivational interviewing techniques	TG	**MI** *WHO Brief Intervention for Hazardous and Harmful Drinking. A Manual for Use in Primary Care* (80) + education on reproductive health	20 min	6 monthly sessions
						CG	Nutrition intervention on nutritional needs for women and their children and women living with HIV	20 min	6 monthly sessions
Mertens et al. (97) ^b^	South Africa	Public-sector clinic in Delft, a township in the Western Cape province	Original study: n=403 (52% female, age range: 18–24)	Individual	Primary care nurse practitioners who received a 3-day training by experienced practitioner and trainer	TG	**MI** *Health Behavior Change: A Guide for Practitioners* (108) + referral resource list for drinking and drug use	10 min	Single session
						CG	referral resource list for drinking and drug use	n/r	n/r
Nadkarni et al. (89)	India	10 primary health centers in Goa	n=377 (all male, age: M=42.0, SD=11.4)	Individual	11 lay counselors recruited from local community (at least secondary school education, selected after an interview, 2 week training, 6-month internship and testing through exam and performance in standardized role-plays)	TG	**MI + CBT-elements** *Counseling for Alcohol Problems* (109) + EUC (consultation with the Primary Health Center physician, provision of the AUDIT screening and of a contextualized version of the WHO Mental Health Gap Action Programme guidelines for harmful drinking)	30–45 min per session (M=42.4 min, range: 40.9-43.7)	1–4 weekly or fortnightly sessions (M=2.8 sessions, 95% CI 2.7–3.0)
						CG	EUC: same as TG	n/r	n/r
Noknoy et al. (105)	Thailand	Primary care unit in rural Northeastern Thailand	n=117 (8.5% female, age: M=37.0, SD=10.0)	Individual	Nurses who received a single 6-hour training session	TG	**MI** *Motivational Enhancement Therapy (MET*): originally developed as a brief four-session adaptation of Motivational Interviewing in Project MATCH (53)	15 min	3 sessions: on day 1, 2 weeks and 6 weeks after baseline assessment
						CG	assessment only	n/r	Single session
Omeje et al. (87)	Nigeria	Infectious disease clinics at 10 community health centers, hospitals & HIV service centers in Enugu State, Nigeria	n=124 (31.45% female, age: M=33.76, SD=2.16, range: 27–56)	Group (sample split into 6 subgroups, no. of P. per sub-group n/r)	University personnel (the study authors), formally trained as counselors and psychologists and expertise in the principles and practice of Rational Emotive Behavior Therapy theory	TG	**Rational emotive health therapy** *Rational emotive health therapy Treatment Manual for Alcohol Use Disorder [RTMAUD*; (110)]	50 min	20 sessions held twice per week for 10 consecutive weeks
						CG	waitlist	-	-
Pal et al. (100)	India	Recruitment within participants of an earlier community-based study (house-to-house survey)	n=90 (all male, age: M=29.7, SD=9.89)	Individual	1 local health worker (social service officer) who delivered both intervention and control conditions (training: n/r)	TG	**MI** *Brief Intervention: A Manual for Practice* (111) based on *“Feedback, Responsibility, Advice, Menu, Empathy and Self-Efficacy”* as critical elements of BI [*FRAMES*; (52)]	45 min	2 sessions separated by a 3–5-d gap
						CG	Simple advice (SA): empathic expression of concern based on consequences, with an advice to cut down or stop alcohol use	5 min	Single session
Papas et al. (112)	Kenya	HIV outpatient clinic in Eldoret, Western Kenya	n=75 (men and women, gender rate n/r, age: M=37.1, SD=8.4,)	Group (12–15 same gender P.)	2 para-professionals meeting certification procedures after trainee program who received 175 and 300 h, respectively, of total training/supervision prior to trial	TG	**CBT-based intervention** *Systematic cultural adaptation of cognitive-behavioral therapy to reduce alcohol use among HIV-infected outpatients in Western Kenya* (113)	90 min	6 weekly sessions
						CG	routine medical care provided in the HIV-outpatient clinic	n/r	n/r
Peltzer et al. (93)	South Africa	42 primary health care clinics in the three provinces with the highest tuberculosis caseload	n=1196 (26% female, age: M=36.7, SD=10.9)	Individual	Lay HIV counselors (to implement the intervention) and nurses (to assist when necessary) from the study clinics who received formal training (lay counselors 3 d, nurses 2 d)	TG	**MI** No manual specified; *Information-Motivation-Behavioral Skills (IMB) Mode*l (114) was used to guide alcohol reduction intervention	15–20 min	2 sessions: on day 1 and within one month after baseline evaluation
						CG	P. received a health education leaflet on responsible drinking	-	-
Pengpid et al. (103)	South Africa	Outpatients of a hospital in Gauteng, Northern South Africa	n=392 (27.6% female, age: M=35.6, SD=n/r)	Individual	Research assistant counselors who received 5 d of training (role playing and general skills training techniques; research assistants were observed in role-play demonstrations until performance criteria are met)	TG	**MI** No manual specified; *Information-Motivation-Behavioral Skills (IMB) Mode*l (114) was used to guide alcohol reduction intervention	20 min	Single session
						CG	P. received a health education leaflet on responsible drinking (no feedback on alcohol-screening)	-	-
Pengpid et al. (104)	Thailand	Four district hospitals in Nakhon Patthom province	n=206 (0.5 % female, age: M=36.8, SD=11.0)	Individual	Research counselors with a university degree in a health related background who received 4 d of training (practical approach, mainly addressed issues deemed essential for implementation of BI in clinic operations)	TG	**MI** BI (integrative for both alcohol and tobacco use): The *ASSIST-linked brief intervention for hazardous and harmful substance use: manual for use in primary care* (81) and *Brief intervention for heavy drinking smokers* by Kahler et al. [treatment provider training manual (115)]	n/r	3 sessions within a period of 3 weeks
						CG1	BI (alcohol use only): (81)	n/r	3 sessions within a period of 3 weeks
						CG2	BI (tobacco use only): (115)	n/r	3 sessions within a period of 3 weeks
Rendall-Mkosi et al. (94)	South Africa	Rural area in the Western Cape province (farms and six primary care clinics)	n=165 (all female, age: M=29.8, range: 18–44)	Individual	Locally recruited and trained lay counselors	TG	**MI** Manual developed by the *CHOICES* (*Changing High-risk Alcohol Use and Improving Contraception Effectiveness Study*) Intervention Research Group (116) + Provision of information pamphlet on FAS prevention and woman’s health	n/r	5 sessions over 2 months
						CG1	Group-based life-skills training intervention: arm not completed due to logistic problems and poor adherence to the intervention	-	-
						CG2	Provision of information pamphlet (same as TG)	-	-
Segatto et al. (88)	Brazil	Three general emergency rooms in Southeastern Brazil	n=175 (17% female, age: M=21.8, SD=2,6 range: 16–25)	Individual	TG: senior psychologist previously trained according to the MI principles CG: 3 trained psychology-students (minimum Bachelor-level)	TG	**MI** No manual specified; Intervention based on: preparing people to change addictive behavior (117) + educational brochure (same as CG)	45 min	Single session
						CG	Provision of a brochure on the risks of alcohol consumption and possible ways to consider reduction, was read by P. and discussed with facilitator	max. 5 min	Single session
Sheikh et al. (90)	Zambia	Chainama Hills Hospital in Lusaka	n=114 (3,5% female, age range: 18–53)	P. with at least one relative	1 psychosocial counselor (training: n/r)	TG	**MI + relative as co-therapist** *WHO mhGAP Intervention Guide for Alcohol Problems* (118) + relative as co-therapist (tasks: providing alternative activities to drinking, help P. to attend follow-up appointments, tell P. to remain abstinent, report to study team if they see signs of relapse) + detoxification with diazepam and vitamin supplem.	20 min	Single session
						CG	detoxification with diazepam and vitamin supplem.	–	–
Shin et al. (92)	Russia	Tomsk Oblast Tuberculosis Services	n=196 (18% female, age: M=40.1, SD=11.2)	Individual	TB physicians who received BI-training (including theoretical framework, specific cultural adaptations, videos of mock MI-sessions; competency assessed using role-plays), training on naltrexone, including the administration, dosing, side-effect management and contraindications	TG1	**MI** *Helping Patients who drink too much: A Health Practitioner’s Guide* (119) adapted to the local context and modified to be implemented in routine TB Services	10–15 min within the standard 45–60-min TB appointm.	6 monthly sessions
						TG2	Administration of Naltrexone (NTX; daily single dose of 50 mg for 6 months) paired with focused intervention (no MI)	5–10 min within the standard 45–60-min TB appointm.	6 monthly sessions
						TG3	**MI** TG1 + TG2	15–25 min within the standard 45–60-min TB appointm.	6 monthly sessions
						CG	TAU: standard referral to and a narcologist only	standard 45–60-min TB appointm.	6 monthly sessions
Sorsdahl et al. (98) ^b^	South Africa	Emergency department in Cape Town	n=335 (34.5% female, age: M=28, range 18–75)	Individual	5 peer counselors who received 18 h of training in MI by a MI-certified trainer (incl. proficiency testing), 3 half-day booster trainings to limit intervention drift, 12 h of training in Problem Solving Therapy (incl. proficiency testing), further training e.g. in substance use and associated risks	TG1	**MI** *The ASSIST-linked brief intervention for hazardous and harmful substance use: manual for use in primary care* (81)	20 min	Single session
						TG2	**MI + CBT elements** *The ASSIST-linked brief intervention for hazardous and harmful substance use: manual for use in primary care* (81) + Problem Solving Therapy (PST; CBT-approach, no manual specified)	20 min per MI-session/45–60 min per PST-session	Single session MI + 4 weekly sessions of PST
						CG	psychoeducation only (brochure providing information on the effects of substance use)	-	-
Wandera et al. (99)	Uganda	Clinic for Infectious Diseases within a public hospital in Kampala	n=337 (34.4% female, age range: 32–46)	Individual	Counselors (minimum bachelor’s degree) with >5 years of experience in HIV (but not alcohol) counseling who received training workshop on treatment administration (including role-play exercises) as well as a treatment manual	TG	**MI** MI (no manual specified) + Standard Positive Prevention counseling (SPP; education on HIV including risks of alcohol use and encouragement to reduce alcohol intake)	30–60 min	Single session
						CG	SPP: same as in TG	10–30 min	Single session
Witte et al. (91)	Mongolia	National AIDS Foundation in Ulaanbaatar, within services for female sex workers	n=166 (all female, age: 9.6% <25 years)	Group (6–8 women)	Female facilitators who received a standardized training	TG	**MI** MI (no MI-manual specified) + HIV sexual risk reduction (HIV-SRR) intervention adapted from a relationship-based HIV/STD prevention program for heterosexual couples (120), incl. alcohol use as a contributing factor to sexual risk	90 min	4 weekly sessions +2 additional MI-sessions
						CG1	HIV-SSR (same as TG)	90 min	4 weekly sessions
						CG2	wellness promotion (focus on relaxation, the importance of exercise and a healthy diet)	90 min	4 weekly sessions

a were information was available, number and professional background of facilitators as well as duration/intensity of training were reported.

b for quantitative analyses, a subsample has been obtained from the author. The characteristics listed here describe the full sample as reported within the publication.

BI, brief intervention; CG, control group; TG, treatment group; EUC, enhanced usual care; TAU, treatment as usual; n/r, not reported; P, participant(s); STI, sexually transmitted infections.

### Intervention Characteristics

#### Lengths and Intensities

All but one study evaluated interventions that qualified as “brief” (1–4 sessions) or “moderate” length (5–7 sessions) following Babor’s (11) definition. With 20 group therapy sessions, the study by Omeje et al. (87) qualifies as “intensive” treatment and thus is by far the study with the highest intervention exposure which must be kept in mind when interpreting the results. For the 17 studies employing individual therapy formats the number of sessions in the treatment groups ranged from one to six sessions with nine interventions consisting of a single session only. Individual sessions lasted between 10 and 60 min, while total intervention exposure time over all sessions ranged from 10 min (97) to approximately 4 h (98). Four trials used group therapy formats (87, 91, 96, 112) with the number of sessions in the treatment groups ranging from 1 to 20 sessions and total intervention exposure time over all sessions ranging from 3 h (96) to 16 h and 40 min (87).

#### Therapeutic Components

The majority of trials (n=18) based their interventions on the principles of MI with most of them explicitly referring to Miller and Rollnick’s MI-rationale (117, 121). MI-based treatments varied with regard to specific techniques, arrangement of components, and intensities. Miller and Rollnick (122) emphasize that MI was not a technique in and of itself and neither was there a step-by-step script to follow when employing it. Accordingly, MI-based interventions were not structured into consecutive components but into communication styles and core skills. However, in order to systemize the elements emerging from the 18 trials using elements of MI, we categorize them into four components comprising the most frequently used topics as well as corresponding techniques. First, the strategy of providing participants with a personalized *feedback* on the result of alcohol related screening and illustrating its meaning was used in the majority of trials [e.g. (89, 91) and all ASSIST-based interventions]. Miller and Rollnick (122) emphasize that personalized feedback was not an essential component of MI, although widely assumed as such. Second, the *provision of structured information about alcohol use* generally plays a major role in all MI-based trials. In all trials, participants in the experimental groups received general psychoeducation about the consequences of alcohol on physical and mental health, either verbally within the counseling setting [e.g. (98)] or in the form of written material [e.g. (88)]. In trials that embedded MI-based treatments into other health programs, the information component was often specific for alcohol consumption in relation to the respective health issue, such as education on how alcohol affects communication skills and sexual behaviors and increases risk for HIV or sexually transmitted infections [STI; e.g. (91, 99)]. The third component is also found in all MI-based trials in this review and includes the elicitation of the participant’s *ambivalence*, *participant-perceived* *importance* of and *confidence* in achieving the behavior change, and the subsequent *enhancement* of all three states. To facilitate these objectives, a variety of structured techniques were employed across the studies with the aim of evoking the participant’s own motivation to reduce drinking and confidence in succeeding (“change talk”). These include (group) discussions about pros and cons of change [e.g. (105)], role-plays to practice how to behave in alcohol-related “high-risk situations” (96), or the explicit recognition of prior attempts to cut down (99). Finally, the fourth component frequently employed across the 18 MI-based trials comprises techniques helping participants to *plan* their behavior change in detail and set specific and appropriate *goals*. Examples are the development of a “habit-breaking plan” (102), helping participants identify their personal aim (e.g. reduced drinking vs. abstinence) and planning measurable goals in changing drinking behavior [e.g. (105)]. Furthermore, Miller and Rollnick (51) emphasize the “cooperative and collaborative partnership between patient and clinician” as a key principle and foundation necessary for the therapeutic skills and styles to have an effect. To account for that, most MI-based studies in this review used expressions such as “client-centered” [e.g. (99)] to describe their facilitators’ general attitude toward the participants, “empathic counseling style” [e.g. (105)] as a method to establish good rapport, and “reflective listening” [e.g. (99)] or “open-ended questioning” [e.g. (89)] as communication styles to be employed by the practitioners.

Among the 18 studies employing an MI-based approach, only six used it as stand-alone intervention without any further additional components (88, 97, 100, 101, 104, 105), while the remaining used the MI-based approach as *one* method and blended it with other treatment components extending beyond the scope of MI. Two studies (89, 98) delivered cognitive and behavioral components within their MI-frameworks, with examples being problem-solving, drink refusal skills training, and handling of peer pressure or difficult emotions. The interventions conducted by Peltzer et al. (93) and Pengpid et al. (103) were informed by the Information-Motivation-Behavioral Skills (IMB) model (114) which integrated behavioral (but not cognitive) elements into the MI-framework, namely “behavioral skills related to preventive actions” (not further specified) (103). One study enhanced the MI-based intervention through administration of the opiate antagonist Naltrexone for the prevention of relapse (92) and one established the active integration of a close family member into the intervention (90). Furthermore, some studies combined their MI-based intervention with non-alcohol-related psychosocial interventions for health topics such as TB (92, 93), reproductive health for female sex workers (102), or HIV (91, 96, 99).

Three trials did not or did not explicitly describe employing elements of MI. One of them was the WHO multinational study that conducted a brief intervention based on a problem-solving model using mainly behavioral techniques (79). The study by Papas and colleagues (112) exclusively applied a cognitive-behavioral approach without explicitly mentioning motivational elements. Finally, in the study by Omeje et al. (87) the authors used an adaptation of Ellis’ rational emotive behavior therapy (123) which, apart from cognitive and behavioral elements, included “emotive” techniques such as satiric songs related to alcohol and other humorous methods with the intention of generating feelings that help to challenge and change dysfunctional attitudes toward drinking. Almost all studies implemented manualized intervention approaches.

#### Intervention Facilitators

Most interventions were conducted by trained lay counselors such as nurses, clinic staff, health workers, or other paraprofessionals, most of them with no or only minimal prior experience in counseling. Some trials formally required the facilitators to have at least a secondary school or a university degree. The majority of studies specified their training procedures. Among those, training intensities ranged widely from 6 h to 300 h or even a 6-month-trainee-program followed by formalized certification procedures (89). Fourteen trials offered weekly, bi-weekly, or monthly supervision to the facilitators, mostly conducted by the project’s principle investigators, clinical supervisors, or peer lay counselors.

#### Comparison Conditions

Apart from a non-alcohol vs. alcohol-focus, the comparison conditions differed greatly regarding their intensities and modalities. Only three studies employed active comparison conditions representing state-of-the-art alcohol interventions (89, 98, 104) and five studies compared the experimental intervention with minimal interventions using the simple provision of advice concerning the reduction of drinking. Other comparison conditions consisted of the treatment considered as “usual care” in the facilities where the study was conducted such as referrals to physicians, the delivery of written material on alcohol consumption, nutrition or wellness promotion interventions, medical detoxification, and assessment-only. For detailed information about the characteristics of intervention and control conditions including manuals and facilitators see **Table 1** as well as **Supplementary Table 1**.

### Duration of Trials and Retention Rates

Across the 21 trials, post-treatment assessment time points of alcohol-related measures were reported referring either to post-enrollment (n=9) or post-intervention (n=12). They were conducted anywhere from 1-month to 12-months post-enrollment or -treatment, with most trials conducting assessments at least two points in time. Most studies assessed participants at either 3 or 6 months after enrollment or intervention. The median reported retention rate was 80% (range: 39%–93%) for 3-months FU-assessments in the experimental groups and 84.5% (range: 54%–97%) in controls. For 6-months FU-assessments the median reported retention rate was 74% (range: 59%–98%) in the experimental groups and 85% (range: 56%–96%) among the control groups. Attrition rates typically increase with trial duration. Interestingly, this was not the case here, since out of the eight studies reporting retention rates for both 3-months and 6-months FU-assessments five studies (95, 96, 99, 103, 105) had higher retention rates at the 6-months FUs compared to the 3-months FUs. The same was true for all three 12-months FU-assessments compared with the 6-months (102, 103) and 3-months (94) FU assessments of the respective studies. See **Table 2** for an overview of trial durations and retention rates for studies included in quantitative analyses.

**Table 2:** Results as reported by the studies included in quantitative analysis.

Study	Outcome Variables directly related to alcohol consumption	Assessment Scales^a,b^(alcohol-related outcome/s only)	FU assessment periods(alcohol-related outcome/s only)	(Narrative) Results as reported in studies(alcohol-related outcome/s only)	Retention in TG and CG	Outcome Variables unrelated to alcohol consumption or non-behavioral outcomes
Assanangkorn-chai et al. (101)	(1) Alcohol consumption in the past 3 months	(1,2) ASSIST-SSIS for alcohol	FU1: 3 months FU2: 6 months (post-intervention)	Sign. reductions in alcohol consumption (1) in both the TG and CG (main effect, but no group x time interaction) Proportions of baseline “moderate-risk” users who had converted to the “low-risk”-category (2) increased sign. over time in both TG and CG (main effect, but no interaction effects)	FU1: 79% (TG), 84% (CG) FU2: 60% (TG), 65% (CG) (P. who did not complete FU1 were not considered for FU2)	Use of other substances in the past 3 months (ASSIST-SSIS)
	(2) Proportions of participants converted from the “moderate-risk” to the “low-risk” category					
Kalichman et al. (95)	(1) Drinking in sexual contexts in previous month	*Single item assessing no. of times they drank alcohol before sex*	FU1: 3 months FU2: 6 months (post-intervention)	P. in the TG reduced their drinking in sexual contexts (1) sign. more than the CG at the 3 months but not at the 6-month follow-up	FU1: 72% (TG), 69% (CG) FU2: 74% (TG), 69% (CG)	Sexual risk and protective behaviors in the previous month (e.g. rate of intercourse occasions protected by condoms) Knowledge about HIV-prevention
L’Engle et al. (102)	(1) Frequency of drinking during the past 30 d	(1–4) *One single item each, ordinal or dichotomous response format*	FU1: 6 months FU2: 12 months (post-enrollment)	Sign. more P. in the TG than in the CG reported reduced drinking in the last 30 d at FU1 and FU2 for frequency of drinking alcohol (1), overall binge drinking (2), binge drinking with paying clients (3), and binge drinking with nonpaying partners (4) Intervention did not impact STI/HIV incidence, condom use, or sexual violence from nonpaying partners but it did sign. decrease sexual violence from paying clients at both FU time-points	FU1: 93% (TG), 94% (CG) FU2: 94% (TG), 95% (CG)	STI-infection or new HIV-positive test result (laboratory-confirmed) Sexual violence victimization and condom use in the last 30 d (assessed separately for paying clients vs. nonpaying sexual partners)
	(2) Binge drinking (=3 or more drinks on the same occasion)					
	(3) Binge drinking before sex during the past 30 d/with paying clients					
	(4) Binge drinking before sex during the past 30 d/with nonpaying partners					
Mertens et al. (97)	(1) Alcohol consumption in the past 3 months	(1,2) ASSIST-SSIS for alcohol *(3) Single item, ‘heavy drinking’ = 3 or more drinks in one occasion (women)/6 or more drinks in one occasion (men)*	FU: 3 months (post-intervention)	Reductions in alcohol ASSIST scores (1) were sign. larger in the intervention arm but prevalence of at-risk alcohol use (2) and heavy drinking (3) at follow-up did not differ across arms	FU: 92% (TG), 88% (CG)	Drug use in the past 3 months (cannabis, methaqualone, cocaine, methamphetamines, inhalants, sedatives, hallucinogens, opiates, and ‘other drugs’; ASSIST)
	(2) At-risk use of alcohol					
	(3) Heavy drinking					
Nadkarni et al. (89)	(1) Remission (AUDIT score of <8)	(1) AUDIT (2–4) TLFB (5) SIP	FU: 3 months (post-enrollment)	CAP (TG) was associated with a sign. higher proportion of abstinence in the past 14 d (3) and remission according to AUDIT 3 months after enrolment (1) compared to the CG No intervention effect on other alcohol-related outcomes (2,4,5) Evidence of a greater intervention effect among those not already trying to change drinking behavior at baseline than among those who had already started to make a change	FU: 87% (TG), 91% (CG)	Serious adverse events (deaths, suicide attempts, unplanned admissions to hospital) Readiness to change Expectations of the usefulness of counseling Economical cost of illness Total days unable to work in the previous month Suicidality Perpetration of intimate partner violence
	(2) Mean daily alcohol consumed in the past 14 d (in grams of ethanol)					
	(3) Percentage of days abstinent in the past 14 d					
	(4) Percentage of days of heavy drinking (definition n/r) in the past 14 d					
	(5) Physical, social, intrapersonal, impulsive, and interpersonal consequences of alcohol					

Noknoy et al. (105)	(1) Amount of alcohol consumption during previous week (drinks/drinking day; hazardous drinking defined as: 14 or more drinks per week or 4 or more drinks per day for men and 7 or more drinks per week or 3 drinks per day for women)	*(1) Four items assessing average drinking and hazardous drinking* *(2*–*6) Items assessing secondary outcome measures* *(all items where embedded into a standardized health survey questionnaire using open and dichotomous response formats)*	FU1: 6 weeks FU2: 3 months FU3: 6 months (post-enrollment)	Self-reported drinks per drinking day (1), frequency of hazardous drinking (1), and of binge drinking sessions (3) were reduced in the TG sign. more than in the CG at FU2 and FU3 (with only little, n.s. attenuation between FU2 and FU3) No consistent evidence of an immediate post-intervention effect at FU1 although self-reported alcohol consumption in both groups fell from baseline to FU3, GGT increased in both groups, raising doubts about the validity of this marker in this sample and/or the validity of the self-reported data in this study	FU1: 85% (TG), 83% (CG) FU2: 93% (TG), 91% (CG) FU3: 95% (TG), 88% (CG)	Serum gamma-glutamyl transferase (GGT), a biological marker available for evaluation of the severity of current drinking, assessed at baseline and FU3 parallel interviews with collateral informants to assess the honesty and accuracy of the information given by the P. (data not reported)
	(2) Consumption of alcohol during the previous month (drinks/week)					
	(3) No. of episodes of binge drinking in the past 7 d					
	(4) No. of episodes of being drunk in the previous month					
	(5) Frequency of accidents and traffic accidents due to alcohol during the previous 6 months					
	(6) Frequency of health care utilization owing to drinking behavior in the previous 6 months					
Omeje et al. (87)	(1) Extent to which P. use alcohol and experience AUD symptoms	(1) AUDS (2) AIBS	FU1: directly post-intervention FU2: two weeks (post-intervention)	Sign. more reduction in alcohol use (1) between TG and CG at post-intervention assessment and at 2-week-FU Sign. more reduction in level of alcohol-related irrational beliefs (2) between TG and CG at post-intervention assessment and at 2-week-FU	FU: 100% (TG), 100% (CG), only P. who attended all group sessions were included into analyses	
	(2) Presence of alcohol-related irrational beliefs					

Pal et al. (100)	(1) Days used alcohol in last 30 d	(1,2, 3) ASI (semi-structured-interview)	FU1: 1 month FU2: 3 months (post-enrollment)	Sign. decrease in alcohol use in the past 30 d (1) as well as the composite score for potential problem areas in substance abusing patients (3) for the group as a whole (main effect) from baseline to FU1 and baseline to FU2 (but not from FU1 to FU2): Decrease in alcohol use in the past 30 d (1) sign. higher for P. in the TG compared to P. in the CG at FU1 and FU2 (+ sign. decrease between FU1 and FU2 in the TG) No change in the problems due to alcohol use (2)	FU1: 97% (TG and CG combined) FU2: 96% (TG and CG combined)	Quality of life in 4 domains: physical, psychological, social, and environmental (WHOQOL) motivation of P. categorizing them to the stage of change regarding pre-contemplation, contemplation, and action stage (RCQ)
	(2) Experienced problems (no. of days; potential problems: medical status, employment and support, drug use, alcohol use, legal status, family/social status, and psychiatric status)					
	(3) Composite measure derived from alcohol use, alcohol use to intoxication, money spent, alcohol problems, being bothered by alcohol problems and the need for treatment in the last 30 d					
Papas et al. (106)	(1) Percent drinking days in the past 30 d (2) mean drinks per drinking day in the past 30 d (in grams of ethanol)	(1,2) TLFB	Post-intervention (6 weeks) FU1: 30 d FU2: 60 d FU3: 90 d (post-intervention) +weekly assessments during treatment phase	CBT was sign. more effective than TAU in reducing reported alcohol use (1,2) at all three follow-up points (with highest effect after 30 d) sign. more CBT than control participants reported abstinence at all follow-ups (e.g. FU3: 69% in TG and 38% in CG)	FU1: 90% (TG), 97% (CG) FU2: 90% (TG), 97% (CG) FU3: 86% (TG), 97% (CG)	withdrawal symptoms (CIWA-Ar)
Peltzer et al. (93)	(1) Change in alcohol-related symptoms in the past 3 months	(1,2) AUDIT (modified to a 3-months-reference period)	FU1: 3 months FU2: 6 months (post-enrollment)	Reductions in AUDIT total score (1) as well as in the number of positively screened P. (2) over time (=more non-drinkers) in both, TG and CG, (sign. main effect, but no intervention effect)	FU1: 39% (TG), 54% (CG) FU2: 59% (TG), 79% (CG)	Successful TB response, classified by WHO as cured or treatment completed
	(2) No. of non-drinkers in the past month (FU2 only)					

Pengpid et al. (103)	(1) Alcohol-related symptoms in the past 6 months	(1) AUDIT (modified to a 6-months-reference period) (2) AUDIT-3 (third item only)	FU1: 6 months FU2: 12 months (post-intervention)	Alcohol consumption (1,2) declined sign. in both TG and CG No intervention effect on total AUDIT score (1) or heavy episodic drinking (2)	FU1: 66% (TG), 56% (CG) FU2: 73% (TG), 71% (CG)	
	(2) Frequency of heavy episodic drinking in the past 6 months					
Pengpid et al. (104)	(1) Alcohol consumption in the past 3 months	(1) ASSIST-SSIS for alcohol (2) TLFB	FU1: 3 months FU2: 6 months (post-intervention)	From baseline to FU1 and FU2 alcohol consumption declined sign. in all groups: the TG (conjoint/polydrug-intervention) and both CGs (single-drug-interventions for alcohol and tobacco) No interaction-effects for alcohol-specific outcomes	FU1: 75% (TG), 74% (CG1), 74% (CG2) FU2: 73% (TG), 73% (CG1), 72% (CG2)	Tabacco consumption in the past 3 months (ASSIST) Tabacco use in the past week (TLFB)
	(2) Quantity of drinking in the past week (in standard drinks but ethanol content n/r)					
Segatto et al. (88)	(1) Pattern of alcohol consumption over the previous 3 months	(1) ACQ (2) RAPI	FU: 3 months (post-enrollment)	Sign. reductions in alcohol abuse (1) and related problems (2) were found in both groups over time (main effect) but no difference between groups was observed	FU: 85% (TG), 85% (CG)	Perception of future risks associated with excessive alcohol use considering that the pattern of alcohol abuse does not change within 3 months (APRA) Motivational stage to change behavior/readiness to change (RCQ)
	(2) Drinking behavior and negative consequences associated with alcohol abuse in the previous 3 months					
Sheikh et al. (90)	(1) No. of days of abstinence following discharge	*(1) One open-response item* (2) AUDIT-C (first three items only)	FU: 8 weeks (post-intervention)	Sign. longer time to first relapse (1) in the TG (M=51 d) compared to the CG (M=10 d) Amount and frequency of alcohol consumption (2) was sign. lower in the TG compared to the CG	FU: 100% (TG), 100% (CG); P. who did not attend FU-appointments were contacted to complete FU over the phone (13%)	“Additional information was obtained from relatives on the participant’s drinking habits” (not reported)
	(2) Amount and frequency of drinking					
Sorsdahl et al. (98)	(1) Alcohol consumption in the past 3 months	(1) ASSIST-SSIS for alcohol	FU: 3 months (post-enrollment for TG1 and CG; post-intervention for TG2)	ASSIST scores (1) sign. decreased from baseline to FU in all three arms Alcohol consumption (1) at FU was sign. lower in the TG2 than in the TG1 and CG (interaction effect) but no difference in alcohol consumption between CG and TG1	FU: 62% (TG1), 42% (TG2), 60% (CG)	Depression (CES-D) Frequency of substance-related injury, physical and verbal violence, and police interaction
Wandera et al. (99)	(1) Quantity of drinking in the past 30 d (grams of ethanol)	(1,4,5) TLFB (2) AUDIT-C (modified to a 3-months-reference period) (3) AUDIT (modified to a 3-months-reference period)	FU1: 3 months FU2: 6 months (post-enrollment)	Sign. overall reduction of alcohol consumption (2) at FU1 and FU2 for both groups but no intervention effect for MI counseling (TG) over positive prevention counseling (CG) MI appeared effective among women only regarding alcohol consumption (2)	FU1: 87% (TG), 89% (CG) FU2: 98% (TG), 96% (CG)	Depression (CESD-10) HIV clinical data
	(2) Alcohol-related symptoms in the past 3 months					
	(3) Proportion of P. with AUDIT score ≥8 points					
	(4) Median number of drinking days in the past one month					
	(5) Average number of alcohol standard drinks consumed on a typical drinking day					
Witte et al. (91)	(1) Alcohol-related symptoms in the past year	(1) AUDIT	FU1: 2 weeks, but data for (1) not collected at FU1 FU2: 3 months FU3: 6 months (post-intervention)	All three conditions sign. reduced harmful alcohol use(1) at FU2 and FU3. No differences in effects were observed between conditions.	FU1: no outcomes of interest assessed FU2: 67% (TG1), 79% (TG2), 73% (CG) FU3: 67% (TG1), 81% (TG2), 85% (CG)	Sexual risk behavior: no. of unprotected vaginal sexual acts with paying clients in the past 90 d

a unstandardized/self-developed scales in italics.

b reference periods (unless otherwise stated): AUDIT=12 months; ASSIST=3 months/ever.

ACQ, Alcohol Consumption Questionnaire; AIBS, Alcohol-related Irrational Belief Scale; APRA, Alcohol Perception of Risk Assessment; ASI, Addiction Severity Index; ASSIST-SSIS, ASSIST-Specific Substance Involvement Scores; AUDIT, Alcohol Use Disorder Idetification Test; AUDS, Alcohol Use Disorder Scale; CES-D, Center for Epidemiological Studies Depression Scale; CESD-10, 10-item Center for Epidemiology Studies on Depression Scale; CG, control group; CIWA-Ar, Revised Clinical Institute Withdrawal Assessment for Alcohol scale; FU, follow-up; no., number; n.s., non-significant; n/r, not reported; P., participant(s); RAPI, Rutgers Alcohol Problem Index; RCQ, Readiness to Change Questionnaire; sign., significant/significantly; SIP, Short Inventory of Problems; STI, Sexually Transmitted Infection; TG, treatment group; TLFB, Timeline Followback; WHOQOL, WHO Quality of Life.

### Risk of Bias

The methodological quality of the included RCTs was variable. For some cases an unfavorable rating has to be put into perspective, since trials were conducted over a 25-year period. An overview of all risk of bias judgments is displayed in **Table 3** and a detailed version including reasons for ratings can be found in **Supplementary Table 2**.

**Table 3:** Risk of bias assessment.

	Selection Bias (sequence generation)	Selection Bias (allocation concealment)	Performance Bias (blinding of subjects)	Detection Bias (blinding of outcome assessment)	Attrition Bias (handling of incomplete outcome data)	Reporting Bias (selective reporting)			
Assanangkornchai et al. (101)	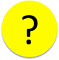	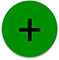	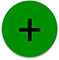	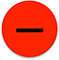	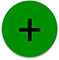	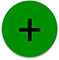		**key**	
Babor et al. (79)	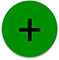	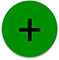	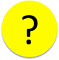	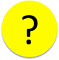	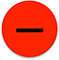	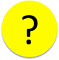		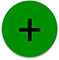		Low risk of bias
Kalichman et al. (95)	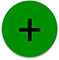	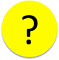	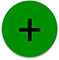	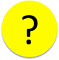	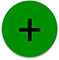	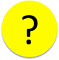		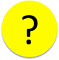		Unclear risk of bias
Kalichman et al. (96)	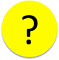	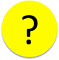	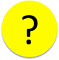	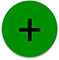	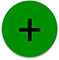	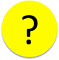		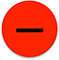		High risk of bias
L’Engle et al. (102)	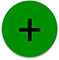	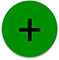	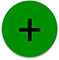	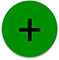	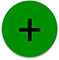	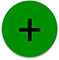			
Mertens et al. (97)	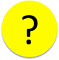	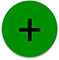	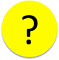	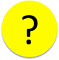	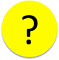	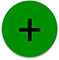			
Nadkarni et al. (89)	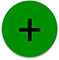	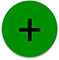	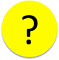	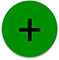	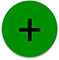	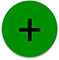			
Noknoy et al. (105)	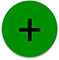	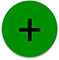	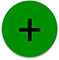	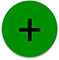	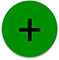	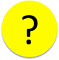			
Omeje et al. (87)	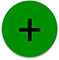	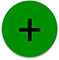	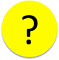	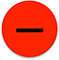	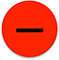	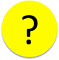			
Pal et al. (100)	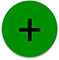	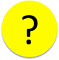	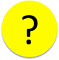	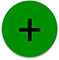	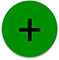	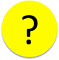			
Papas et al. (112)	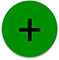	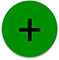	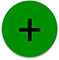	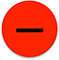	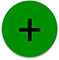	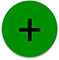		
Peltzer et al. (93)	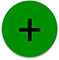	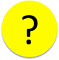	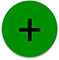	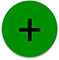	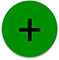	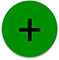		
Pengpid et al. (103)	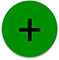	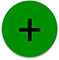	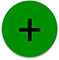	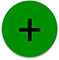	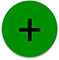	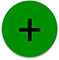		
Pengpid et al. (104)	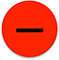	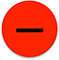	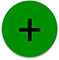	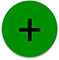	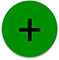	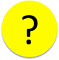		
Rendall-Mkosi et al. (94)	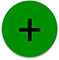	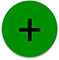	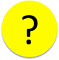	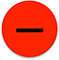	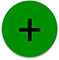	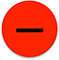		
Segatto et al. (88)	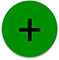	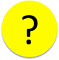	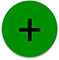	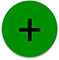	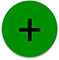	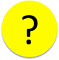		
Sheikh et al. (90)	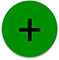	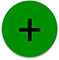	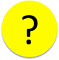	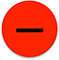	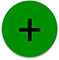	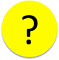		
Shin et al. (92)	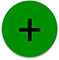	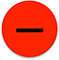	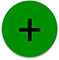	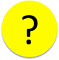	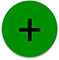	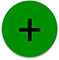		
Sorsdahl et al. (98)	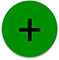	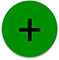	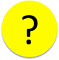	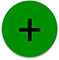	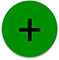	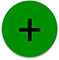			
Wandera et al. (99)	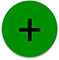	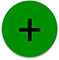	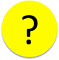	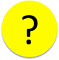	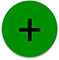	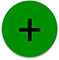			
Witte et al. (91)	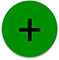	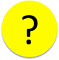	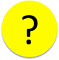	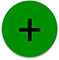	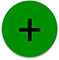	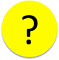			

### Outcome Measures

The included studies employed a wide variety of outcome measures. Effects were reported on more than 30 different alcohol-related outcome variables, including measures of frequency, amount and patterns of alcohol consumption, alcohol-related symptoms, binge drinking occasions, dependency symptoms, alcohol-related irrational beliefs, and problems resulting from drinking. These were measured using about 20 different assessment instruments including standardized clinical screening tools, timeline-followback interview assessments (TLFB), and self-developed scales. The most frequently employed instruments were the AUDIT and the ASSIST, both measuring frequency, amount and patterns of alcohol consumption, and alcohol-related symptoms. For details on measured outcome variables and applied instruments see **Table 2** and **Supplementary Table 1**.

### Main Results of Quantitative Analyses: Effect Sizes 

Among the 17 studies included in the quantitative analyses 11 reported outcomes from the category *alcohol-related symptoms*, five from the category *alcohol* *amount consumed per defined unit*, and three studies reporting outcomes from both main categories. Four studies reported none of the two measures and therefore entered analyses with other alcohol-related outcomes (see **Table 4** for details). In terms of assessment time points, 14 studies reported a 3-month FU and nine studies reported 6-month FU measurements, and eight studies reported both. Only two studies did not assess outcomes at any of the two time points and therefore entered analyses with their 2-month (90) and 2-week (87) FU assessments instead. Enhanced treatment conditions relevant for this review were conducted only by Sorsdahl et al. (98) and the respective results of this condition were utilized for effect size calculations. Positive effect sizes indicate that the intervention was more effective than the control condition in reducing alcohol-related symptoms and vice versa for negative effect sizes. All effect sizes are displayed in **Table 4**.

**Table 4:** Effect sizes.

Study	Intervention in TG	Dependent variable	Effect sizes for *3-months* post assessments			Effect sizes for *6-months* post assessments				Type of analysis
			Within-condition for TG	Between-condition for TG vs. alc.-unrelated CG	Between-condition for TG vs. alc.-related CG	Within-condition for TG		Between-condition for TG vs. alc.-unrelated CG	Between-condition for TG vs. alc.-related CG	
**MAIN OUTCOMES**			**Hedge’s g_av_**	**Hedge’s g_s_**	**Hedge’s g_s_**		**Hedge’s g_av_**	**Hedge’s g_s_**	**Hedge’s g_s_**	
**(1) Alcohol-related symptoms**										
Nadkarni et al. (89)	MI + CBT	AUDIT	0.65	–	0.12	–		–	–	Intent-to-treat ^a^
L’Engle et al. (102)	MI + RH counseling	AUDIT	–	–	–	1.78		0.60	–	Complete case ^a^
Wandera et al. (99)	MI + HIV counseling	AUDIT (modified to 3-months reference period)	0.65	–	-0.19	0.64		–	-0.04	Intent-to-treat
Pengpid et al. (104)	MI	ASSIST-SSIS for alcohol	2.07	0.17 ^b^	–	2.53		0.55 ^b^	–	Intent-to-treat
Pengpid et al. (103)	MI + behav. elements	AUDIT (modified to 6-months reference period)	–	–	–	1.42		–	-0.15	Intent-to-treat
Witte et al. (91)	MI + HIV counseling	AUDIT	1.26	-0.46	-0.20	1.50		-0.36	-0.39	Intent-to-treat
Sorsdahl et al. (98)	MI + CBT	ASSIST-SSIS for alcohol	2.15 ^c^	–	0.83 ^c^	–		–	–	Complete case ^d^
Peltzer et al. (93)	MI behav. elements	AUDIT (modified to 3-months reference period)	1.82	–	-0.17	2.46		–	0.23	Intent-to-treat
Assanangkornchai et al. (101)	MI	ASSIST-SSIS for alcohol	1.83	–	0.13	2.54		–	0.19	Intent-to-treat
Mertens et al. (97)	MI	ASSIST-SSIS for alcohol	1.20	–	0.06	–		–	–	Complete case ^d^
Sheikh et al. (90)	MI + relative as co-therap.	AUDIT-C	3.61 ^e^	2.15 ^e^	–	–		–	–	Complete case
**(2) Alcohol amount consumed per defined unit**										
Nadkarni et al. 2017 (89)	MI + CBT	Mean standard drinks per day (past 14 d)	–	–	0.17	–		–	–	Intent-to-treat
Papas et al. (112)	CBT	Mean standard drinks per drinking day (past 30 d)	0.96	0.40	–	–		–	–	Complete case
Noknoy et al. (105)	MI	Mean standard drinks per drinking day (past 7 d)	0.66	0.62	–	0.80		0.52	–	Intent-to-treat
Wandera et al. (99)	MI + HIV counseling	Mean standard drinks per drinking day (past 3 months)	-0.06	–	-0.27	-0.07		–	-0.10	Intent-to-treat
Pengpid et al. (104)	MI	Past week alcoholic use units	0.79	0.20 ^b^	–	0.87		0.10 ^b^	–	Intent-to-treat
**(3) Other outcomes**										
Kalichman et al. (95)	MI + HIV counseling	No. of times of alcohol use before sex (past month)	0.49	0.36	–	0.29		0.34	–	Intent-to-treat
Pal et al. (100)	MI	Number of d used alcohol (past 30 d)	1.58	–	0.87	–		–	–	Complete case
Omeje et al. (87)	Rational-Emotive Th.	AUDS ^f^	7.42 ^g^	5.85 ^g^	–	–		–	–	Complete case
Segatto et al. (88)	MI	mean no. of alcohol use days (past 3 months)	0.45	–	-0.13	–		–	–	Complete case

a data of relevant outcome was provided from authors upon request.

b Hedge’s g_s_ value is based on a comparison of CG1 (alcohol use only) and CG2 (tobacco use only) since an integrative treatment condition (tobacco + alcohol) was not of interest here.

c Displayed is the effect size value of TG2 (enhanced condition). Effect size values for TG1 are: g_av_=1.17 and g_s_=0.05 for 3-months FU assessments.

d data of relevant subsample was provided from authors upon request.

e FU-assessment conducted at 2-months, not 3-months, post intervention (no later FU-assessment available).

f Alcohol Use Disorder Scale; ad-hoc developed scale; only limited information available about it, therefore categorized in “other outcomes”.

g FU-assessment conducted at 2-weeks not 3-months, post intervention (no later FU-assessment available).

“—”indicates that effect size was not calculable.

Negative effect sizes indicate superiority of the comparison group over the respective experimental group on this particular measure.

Hedge’s g_av_ (within-condition effect sizes) uses the average standard deviation of both repeated measures as a standardizer.

Hedge’s g_s_ (between-condition effect sizes) uses the pooled standard deviation of both independent measures as a standardizer.

ASSIST-SSIS, ASSIST-Specific Substance Involvement Scores; AUDIT, Alcohol Use Disorder Identification Test; AUDS, Alcohol use disorder scale (ad-hoc developed scale); CG, Control Group; RH, reproductive health; TG, Treatment Group.

#### Alcohol-Related Symptoms

Among the 11 intervention trials which reported an outcome from the category alcohol-related symptoms, all 11 within effect size values (Hedge’s *g_av_*) indicated positive effects for the treatment conditions with the majority of effect size values being larger than *g_av_*>1.0. This was the case for both, 3-months (range: 0.65–3.61) and 6-months (range: 0.64–2.54) FU-assessments compared to baseline.

Between-group effect sizes (Hedge’s *g_s_*) comparing the focus intervention with an *alcohol-unrelated control group* (n=3 studies) ranged widely for the 3-months FU-assessment. We found a superiority of the control group for Witte et al. (91) (*g_s_*= −.46), no effect for Pengpid et al. (104) (*g_s_* = .17), and a very large between-group effect for Sheikh et al. (90) (*g_s_* = 2.15). In the case of Pengpid et al. (104), however, the intervention effect increased toward a medium sized effect measured on the ASSIST scale 6-months post-intervention (*g_s_* = .55).

Comparisons with *alcohol-related control groups* revealed negative to small between-condition effect size values at 3-months (n = 6; range: −.20 to .15) and 6-months (n = 5; range: −.39 to .23) FU-assessments with the highest value (*g_s_* = .23) calculated for Peltzer et al.’s (93) intervention at their 6-months FU.

#### Alcohol Amount Consumed Per Defined Unit

Based on five RCTs that measured the amount of alcohol consumption per defined unit within-condition effect sizes ranged from *g_av_* = −.06 to 0.96 at 3-months FU and from *g_av_* = −.07 to .87 at 6-months FU.

The studies comparing their interventions to alcohol-unrelated control conditions found small to medium between-condition effect sizes (range: 0.20–0.62) at 3-months FU-assessment. The highest effect was calculated for Noknoy and colleagues (*g_s_* = 0.62) (105). The effect remained notable at 6-months FU-assessment (*g_s_* = 0.52).

The two RCTs contrasting their interventions against alcohol-related comparison conditions at 3-months (89) and 3- and 6-months FU-assessments (99) found either no group x time interaction effects (*g_s_* = 0.17) (89) or depicted a negative effect representing the superiority of the respective control group (*g_s_* = −0.27) at 3-months FU-assessment that subsided at 6 months (*g_s_* = −0.10) (99).

#### Other Outcomes

Four studies employed alcohol-related endpoints that could not be allocated to either of our two main outcome categories (87, 88, 95, 100). Calculations of within-condition effect sizes yielded a range from *g_av_* = 0.45 to 1.58 at 3-months FU. For the study conducted by Omeje et al. (87), only a 2-week FU assessment was available, producing a very large within-condition effect size-value of *g_av_*= 7.42 on a self-developed AUD scale. Of these studies, only Kalichman et al. (95) provided data at the 6-months FU point with an effect size value of *g_av_* = 0.29 for number of times of alcohol use before sex in the past month.

For the two studies that compared their intervention to an alcohol-unrelated control condition, we calculated small, but stable between-condition effect sizes of *g_s_* = .36 (3-months FU) and *g_s_* = .34 (6-months FU) for Kalichman et al. (95) and a very large effect of *g_s_* = 5.85 (2-weeks FU) for Omeje et al. (87). For the two studies with alcohol-related comparison conditions we found no effect for Segatto et al. on their dependent variable mean number of alcohol use days in the past 3 months (88), but calculated a large effect size value of *g_s_* = .87 based on the results of Pal et al.’s study at the 3-months FU-assessment (100).

#### Trends of Effect Size Values Across Multiple Assessment Points

Among studies where we were able to calculate between-condition effect size values for two FU-assessments (n=7 studies), the effect increased from FU1 to FU2 for Pengpid et al. (104) (small effect to medium effect) and Peltzer et al. (93) (negative effect to small effect) both reporting on alcohol-related symptoms. The effect size decreased from a small effect to no effect for the second outcome of Pengpid et al. (104). For the other studies the effect remained within the same Cohen’s *d* category (91, 95, 99, 101, 105).

### Narrative Results and Studies Without Numeric Data

As part of the qualitative analysis, we extracted the results as reported within the study publications in addition to calculating effect sizes. All narrative results as reported by the 17 studies included in quantitative analyses can be found in **Table 2**. For four studies (79, 92, 94, 96) we were unable to calculate effect sizes as the necessary information was neither provided within the publications nor after contacting the authors. Due to the lack of numeric data the results of these four studies are only summarized as reported by the authors in **Supplementary Table 3**.

## Discussion

### Summary of Main Results

The present systematic review identified 21 randomized-controlled trials conducted in 15 different LMIC. Generally, methodological quality of the included studies was found to be adequate. Studies included female and male participants who screened as hazardous, harmful, or potentially dependent drinkers. The treatments were predominantly brief interventions provided by trained paraprofessionals in primary care settings. Four studies provided group therapy while all others conducted individual therapy sessions and there was great variation in total intervention exposure and primary outcome measures between the trials. In order to facilitate comparability regarding the effectiveness of interventions we calculated effect-sizes for the 17 studies where sufficient information was available using Hedge’s *g_av_* for within the respective treatment condition and Hedge’s *g_s_* for between-condition effects.

#### Effect Sizes

The majority of within-condition Hedge’s *g_av_* -values were ≥.8 at both 3-months (n=10 out of 17 effect size values) and 6-months (n=8 out of 11 effect size values) FU assessments. From this perspective, the vast majority of interventions had a large to very large positive impact on alcohol-related outcomes. However, looking at between-condition effect sizes, this impression is relativized as Hedge’s *g_s_* -values were generally much smaller. Large and very large effects occurred only in four studies (87, 90, 98, 100) and only at the short-term FU assessment point. What is striking about the studies by Sorsdahl et al. (98) and Pal et al. (100) is that their interventions achieved large between-condition effect sizes although contrasted with alcohol-related comparison procedures. Pal et al. (100) employed an MI-based approach as stand-alone intervention against routine medical care while, in the case of Sorsdahl et al. (98), only the condition in which ASSIST-linked-MI was enhanced with Problem Solving Therapy (an additional CBT component) yielded a differential effect over control (psycho-education only) while ASSIST-linked-MI alone did not. The extremely large between-condition effect size values found by Omeje et al. (87) (*g_s_* = 5.85) and Sheikh et al. (90) (*g_s_* = 2.15) have to be put into perspective. In addition to having methodological limitations such as high risk of attrition bias (all participants who missed one or more therapy sessions were excluded from analyses) and a self-administered ad-hoc developed scale as the primary outcome, Omeje et al. (87) employed the intervention with by far the highest treatment exposure of 20 group sessions of rational emotive health therapy and compared it to a waitlist control group condition. On top of that, the authors chose a very short period of 2 weeks for their only FU assessment. In Sheikh et al. (90), although formally stating only one therapeutic contact of 20 min, the very intensive involvement of the participants’ relatives as co-therapists designated to monitor the participant’s consumption as well as the option to seek support from the study team in case of relapses extend the intervention exposure far beyond the actual counseling session. Moreover, the control group received medical detoxification only.

For the longer-term FU time point, 11 studies reported data after 6 months with only three achieving medium-sized between-condition effects (Pengpid et al. (104) using ASSIST-linked-brief intervention, L’Engle et al. (102) using WHO Brief Intervention for alcohol use, and Noknoy et al. (105) using motivational enhancement therapy). All three studies compared their interventions to alcohol-unrelated control conditions. In summary, among the 17 studies that entered quantitative analyses only six revealed small to medium between-condition effect size values 6 months later. An additional five reported medium to large between-condition effect sizes 3 months after intervention but did not assess subjects at later time points. Our results suggest a modest impact of psychosocial interventions for alcohol-related problems in LMIC.

#### Comparison Conditions

Continuing to consider the nature of comparison conditions to further disentangle the results, it is notable that among the 10 studies with at least small between-condition effect-size values in favor of the experimental group, only three employed active control groups with an alcohol-focus (93, 98, 100). For the remaining seven studies with at least small Hedge’s *g_s_* values the effect sizes corresponded to a comparison between intervention and alcohol-*un*related control group procedures, such as routine medical care, mixed content educational components, or assessment only. Moreover, looking at the seven studies where between-condition effect size values were below .20 or negative, all of them employed alcohol-related comparison groups. Effect size results therefore suggest that as soon as the topic of alcohol is addressed in the control group procedure (e.g. expression of concern about drinking habit and simple advice to cut down, verbal or paper-based information/ psycho-education) there appears to be little to no difference between the conditions regarding their potential for reducing harmful alcohol use in participants. In general it seems that, at least within the rather short re-assessment interval of up to 6 months as investigated in the current review, many different control group procedures (including assessment-only) can lead to consumption-related behavior change.

Similarly, previous reviews on alcohol-focused interventions in HIC [e.g. (56, 61)] have also identified significant reductions in alcohol consumption in assessment-only or minimal-treatment (e.g. simple advice) control groups. Factors that have been postulated to possibly contribute to change in control groups are regression to the mean (124), the *Hawthorne effect* (change in behavior because subjects know they are being studied), and reactivity to assessment [e.g. (58, 125)]. The latter appears especially plausible for short interventions because their assessment processes often last longer than the actual counseling session and similarly focus on alcohol and effects of drinking. In addition, assessments in populations with low education and literacy rates, as are often found in LMIC, are usually done via one-on-one interview. This entails that the mere setting can resemble an intervention and therefore may (unintentionally) actuate unspecific effect mechanisms such as the therapeutic relationship, increase of attention on the problem, or evocation of motivation e.g. via feelings of guilt or remorse; especially since alcohol use and its effects are addressed during the assessment.

#### Interventions

Taking a closer look at the employed interventions, among the 10 studies with at least small positive between-condition effect size values eight employed at least one component of MI (90, 93, 95, 98, 100, 102, 104, 105). The two non-MI studies were the study by Omeje et al. (87) (rational emotive health therapy) which has methodological problems (see above) and Papas et al. (112) who conducted a CBT-based multi-session group intervention. Although promising, we are unable to draw conclusions regarding the effectiveness of pure CBT-interventions in LMIC from only one study. On the other hand, among the seven studies where between-condition effect-size values were below .20 or negative, all employed a MI-component as well (88, 89, 91, 97, 99, 101, 103). Taken together, the current evidence regarding MI-based interventions in LMIC is by far not as clear as one would expect considering its wide-spread use in therapeutic practice and scientific trials in LMIC settings. In sum, MI-based approaches or enhanced MI-based approaches were almost as often not effective as they were effective. This finding, however, matches conclusions drawn from comprehensive reviews summing up data from high income settings [e.g. (57)]. The authors similarly failed to find compelling evidence to support one psychosocial treatment targeting alcohol abuse over another. Nevertheless, some conclusions and recommendations concerning the features of interventions for practice and future study can be derived based on the current findings.

### Properties of Effective Interventions

The heterogeneity of the studies’ settings and populations complicates the process of identifying intervention elements and properties that seem to be associated with positive outcomes regarding alcohol consumption for a majority of examined individuals. Starting with the framework conditions, evidence regarding the most effective dose of intervention input is inconclusive. One review that investigated alcohol-focused interventions conducted in primary care settings in predominantly high-income countries found brief multi-contact interventions to be more effective than other intensities, including brief single contact interventions (126). This matches the evidence found in the current review. Among the eight studies that conducted brief multi-contact interventions of 2–6 sessions, six showed an effectiveness of their experimental group in terms of between-condition effect sizes (93, 100, 102, 104, 105, 112), while only two did not (89, 91). For single-session interventions the ratio was nearly opposite with only two studies showing effectiveness of their interventions over the respective control condition (95, 98), while five did not (88, 97, 99, 101, 103). This is again excluding the trials by Sheikh et al. (90) and Omeje et al. (87), whose interventions appear to be extremely effective, but contain characteristics and methodological issues making them less comparable to all other studies.

Other reviews such as Moyer et al. (62) or Kaner et al. (61) who compare brief alcohol interventions in treatment-seeking and non-treatment-seeking populations or in primary care settings, respectively, argue in favor of brief interventions by concluding that there was no significant advantage of more extended treatments over shorter and even single-session inputs on alcohol reduction. In the current review among the 17 studies with calculable effect sizes eight studies had overall treatment exposures of <45 min. For those eight studies, the ratio of effective vs. non-effective interventions was 3:5 while it was 7:2 for the studies with overall treatment durations of ≥45 min making the longer interventions appear more effective. However, except Omeje et al. (87), practically all of the studies within this review can still be considered short-term interventions as the trials with the longest overall individual counseling exposure reported 120 (102) and 150 min (89). The finding that the majority of these interventions were effective is an important message, especially for low-resource settings within LMIC, where extensive treatment programs may not be a realistic option.

Another aspect that needs consideration is the delivery mode of the interventions. While the authors of the four studies that employed group-based interventions (87, 91, 96, 112) did not explain the reasoning behind their decision for the format, the most obvious factors appear to be cost- and resource efficiency and practicability. Whereas there is some quite promising evidence on CBT in groups for the treatment of hazardous drinking [e.g. (127, 128)], only very limited data can be applied to the question of whether group-delivered MI is effective. The few researchers who have attempted to illuminate the topic have named potential benefits and mechanisms such as direct feedback through peers (129) or positive reinforcement of behavior change by group members (130), however, they remain hesitant to recommend group-based MI and argue in favor of a combined approach (group + individual) instead. Review-level data investigating delivery mode of MI as a moderator is scarce with the existing literature yielding no statistically significant differences between group and individual facilitation among the small number of primary studies available (131). In the present review, a benefit of the group-based approach compared to active control was found for both CBT-infused interventions (87, 112) and for one (96) out of the two MI-based interventions. In any case, given the many potential advantages of group therapy programs for at-risk drinkers in LMIC this therapy modality merits more research attention.

It is fairly difficult to distill the active components of the interventions that have demonstrated good effectiveness, as the great majority of trials in this review used approaches based on MI. However, stand-alone MI-based approaches were rarely used, reflecting the circumstance that combining MI-elements with other interventions for hazardous drinking, usually behavioral or cognitive-behavioral in nature, has become quite common in HIC-settings and this seems to have been transferred to LMIC-settings. By focusing in detail on one research project which has combined MI with CBT, Moyers and Houck (132) have reflected on this widespread practice. They found that combined treatments produce outcomes that are often, but not always, superior to “pure” MI and state that the common rationale for combining MI with other treatment approaches was to help engage patients into the more complex CBT techniques. In the current review, including only the trials that entered quantitative analyses, three out of six MI-only interventions (100, 104, 105), both CBT-only interventions (87, 112), and two out of the four MI+CBT-interventions (93, 98) produced at least small between-condition effect sizes. Only Sorsdahl et al. (98) directly compared their MI-based intervention with the same MI-based intervention blended with CBT and found the combined treatment condition to be by far superior (*g_s_* = .83 for the MI+CBT intervention compared to *g_s_* = .05 for the MI-only condition). However, for Pal et al.’s study (100) an about equally high between-condition effect size value of *g_s_* = .87 was calculated for their two-session MI-based intervention *without* any CBT enhancement. Hence, even when attaching more weight to the studies by Sorsdahl et al. (98) and Pal et al. (100) (which seems legitimate as their interventions were the only ones yielding large between-condition effect size values when contrasted to alcohol-related comparison procedures), we end with a draw between MI-only and a CBT-blended MI-based intervention. Taken together, based on the trials included in the present review, the question of whether MI-only, or enhanced MI-based approaches should be first-line interventions to reduce alcohol-consumption and related symptoms in LMIC-settings cannot be conclusively answered.

The reduction in drinking among controls as found in many studies in the present review [e.g. (91, 93, 101)] might support the conclusion that mere FU could be recognized as a factor favoring change; a phenomenon which has already been recognized decades ago [e.g. (133, 134)]. As an implication for LMIC-settings it can be derived that beneficial effects might already arise from low-threshold, yet mandatory arrangements such as regular brief assessments of individuals combined with continuous communication and psychoeducation regarding alcohol use which could be pragmatically integrated into health care or other community-based services.

On a related note, monitoring seems to be a helpful mechanism to maintain behavior change as demonstrated in the study by Sheikh et al. (90) where a close relative, such as a participant’s spouse, was designated as co-therapist whose task it was to support the study participant’s abstinence (e.g. by helping him/her to avoid places where alcohol is available) and to arrange an additional appointment with the study team if they observed any signs of relapse in the patients. Apparently, this strategy was successful with remarkable results regarding effectiveness (Hedge’s *g_s_*=2.15) and retention (100%) at 8 weeks post-intervention. However, an intervention that interferes with the patient’s social system in a way that dysfunctional processes may eventuate seems ethically concerning, especially without controlling or studying these during or beyond the study period. There are similarities with the community reinforcement approach [CRA (135)] that also acknowledges the role of the social environment in the treatment of alcohol problems, or Behavioral Couples Therapy [BCT (136)] that involves both, the partner and the help-seeking individual into treatment. However, while in BCT and CRA the relationship to the spouse or relative(s) is closely monitored, in Sheikh et al.’s (90) intervention there is no mention about any specialized training or supervision for the relatives who act as co-therapists. Taken together, therapeutic techniques systematically utilizing social normative influence by partners, relatives, or significant others seem to offer some potential regarding the reduction of alcohol consumption in certain constellations. When employing such techniques, supervision structures for individuals within the patients’ social contexts who are involved in the intervention provision have to be considered.

In terms of facilitators the majority of interventions in this review (67%) were conducted by trained and supervised paraprofessionals. The practice of lay counselors conducting mental health interventions is backed up by an emerging number of researchers arguing that common practice elements such as MI-based counseling, psychoeducation, and even more complex techniques such as cognitive restructuring can be taught to personnel trained in other professions such as primary care staff who can then effectively apply them and improve the coverage in settings where no formal mental health infrastructure exists (73, 137, 138).

### Recommendations for Future Research

Given the inconclusive picture we attained regarding the effectiveness of components used in alcohol-focused interventions in LMIC, dismantling trials and dissemination studies seem warranted in order to determine the differential effectiveness of the specific treatment elements.

A rather complex issue emerging from epidemiological research in LMIC is the topic of gender, with differential findings for prevalence rates. This includes generally higher rates found in men [e.g. (24, 139)], differences in drinking motives [e.g. coping motives predicting average alcohol intake for dependent women, but not men (140)], and psychiatric comorbidities [e.g. relationship between comorbid PTSD and alcohol dependence for men but not women (141)]. These findings appear to indicate that interventions should also be gender-sensitive. In most studies in this review, the vast majority of participants were male, with only three studies investigating all-female cohorts and only one mixed-sample study with more women than men. Among these four, two studies found their interventions to be effective (96, 102). Wandera and colleagues (99), whose sample was mixed but predominantly male, found their intervention to be effective for female participants only. Furthermore, female samples examined by the RCTs included in the present review were often extraordinary groups, such as female sex workers (91, 102), possibly due to the fact that hazardous drinking in LMIC is generally more prevalent in males. Hence, such samples may entail some particular risk factors and treatment approaches not best suited for women from the general population (e.g. particular emphasis on alcohol use in the context of HIV risk). Overall, sex-specific evidence in this review is inconclusive as most studies either did not report any gender-specific outcomes at all or were unable to examine whether findings differed by gender or not due to excessive gender imbalances in their cohorts [e.g. (97, 101, 105)]. With regard to the gendered nature of alcohol-related problems in LMIC, prospective alcohol intervention research should account for gender differences and power their samples accordingly.

Moreover, in order to more holistically capture the impact of interventions against excessive alcohol consumption, future trials in LMIC should consider including socially and systemically relevant outcomes, such as the impact of male drinking on intimate partner violence, violence against children, and other adverse effects on families such as stigmatization. In their RCT conducted in India, Satyanarayana et al. (142) found that an integrated cognitive-behavioral intervention targeting IPV perpetration among alcohol-dependent men was able to reduce violence against women and even improve mental health outcomes among participants’ wives and children in addition to having positive effects on alcohol-related problems. While the exacerbation of domestic violence with the involvement of alcohol is also found in HIC, women’s economic dependence and limited social opportunities relative to men are much more pronounced in LMIC and limit the viability of women exiting relationships in which alcohol consumption and violence become excessive (143). To date, research investigating the effectiveness of integrated treatment programs on both outcomes is optimistic but mostly HIC-based [e.g. (144)]. Therefore, the assessment of domestic violence in future alcohol intervention research in LMIC would be of particular importance and may document effects of the interventions beyond the reduction of AUD-symptoms.

None of the studies matching our inclusion criteria conducted their interventions in a conflict or post-conflict setting, which is in line with statements by authors of recent reviews [e.g. (18, 19)]. Therefore, we are unable to draw conclusions about intervention implementability and success in this particular context. However, research in populations affected by conflict points out some contextual factors that are likely to be relevant when trying to provide alcohol interventions in conflict or post-conflict settings. These include higher levels of traumatic exposure, higher prevalence rates of mental health disorders [e.g. (145)], and most likely also a higher density of daily stressors compared to more stable settings (e.g. impoverishment, ongoing insecurity, and impaired social cohesion within communities). Ezard, Debakre, and Catillon (146), who piloted an alcohol-focused brief intervention in a refugee camp in Thailand, additionally noted a “pervasive sense of hopelessness and dispossession” that potentially limited people’s motivation toward behavior change and therefore might have prevented positive treatment outcomes. Given that alarming prevalences of risky alcohol use in conflict-affected civilian populations have been reported (18–20) and given the empirically robust link between alcohol use and aggression [e.g. (42, 45)] it seems crucial to extend the current body of intervention research toward conflict and post-conflict societies.

As a further step beyond the efficacy of interventions, future studies in LMIC should also take important public health issues such as barriers to treatment access, cost-effectiveness, and long-term effects into consideration when evaluating interventions as those are factors relevant for the dissemination and routine-implementation of treatment programs.

### Limitations of the Included Studies

The studies included in this review have several limitations regarding their designs and contents: First, FU periods were rather short, with only three studies reporting FU assessments later than 6 months posttreatment. Consequently, for most interventions no conclusions about their long-term effectiveness could be drawn. Future studies should consider longer FU periods as well as multiple assessment points in order to more clearly measure the sustainability of treatment effects. Second, attrition rates were quite high in some trials which may have limited the studies’ power to detect between-group differences. Future intervention studies in LMIC contexts are advised to allocate more effort and resources toward developing FU procedures which better match the specific setting of the target population. For instance, instead of relying on phone calls researchers could consider the use of local partners to collect detailed location descriptions of the participants’ homes in the event of informal living conditions to reduce attrition between the end of treatment and FU. Third, almost all studies reviewed here relied solely on self-report outcome data which can bring along problems such as memory effects or social desirability, with the latter being prone to variation by group allocation, especially in non-blinded trials. Experience with objective measures in LMIC-contexts is lacking. Consequently the use of measures of recent alcohol consumption in the form of breathalyzers or biomarkers, as well as collateral informants in an attempt to validate self-report-measures should be considered where feasible. Fourth, even though all the trials were RCTs, risk of bias assessment revealed some potential threats to internal validity, such as unclear or deficient sequence generation in four studies, unclear or inadequate allocation concealment in eight studies, and either unclearly or non-blinded outcome assessment in nine studies. However, most RCTs were rather strong on external validity, since they were conducted in naturalistic settings. This includes the choice of locations for service provision, such as primary health centers or hospitals, and the choice of personnel. Fifth, not all studies employed manualized interventions. For those studies this limits the possibility of monitoring treatment fidelity as well as replicability. Sixth, 14 trials in this review (87, 88, 90–100, 112) either included alcohol-dependent individuals only, reported a high proportion of dependent drinkers within their sample, or did not actively attempt to exclude alcohol dependent individuals, while the remaining seven studies did. Although, traditionally, hazardous and harmful, yet non-dependent drinkers have been the target for alcohol brief intervention research, the assumption that the interventions might not be effective in alcohol-addicted individuals has been questioned [e.g. (63, 147, 148)]. In this review, only two out of the 21 studies statistically controlled for a potential moderating effect of baseline drinking level on their outcomes: Nadkarni et al. (89) who included participants with an AUDIT score between 12–19 found no evidence of effect moderation by baseline AUDIT score; and Kalichman et al. (96), whose only inclusion criterion was “alcohol consumption in the previous month”, noted that those who were at the least risk for problem drinking demonstrated the greatest reductions in alcohol use. As these two studies either excluded dependent drinkers (89) or potentially included an unknown number of dependent drinkers (96) we are unable to clarify the debate of whether or not dependent drinkers in LMIC can statistically benefit from alcohol-focused brief interventions. However, among the 10 studies in the current review with effect sizes above .20 only three excluded dependent drinkers from their interventions (102, 104, 105), while all others did not explicitly do so. This suggests that brief alcohol interventions can also be effective when alcohol-addicted individuals are among the participants. Future studies should differentiate more clearly between persons with risky non-dependent drinking patterns and persons with alcohol dependence using reliable measurements. Such a classification would allow for differential analyses on the effectiveness of interventions.

Seventh, it is concerning that, among the 14 studies in this review that potentially or intentionally included alcohol-dependent individuals, only two (94, 98) reported that appropriate measures were in place in the event a participant experienced serious withdrawal symptoms such as seizures, deliria, or other medical complications. Accordingly, the availability of medical staff on site or at least the potential to quickly refer patients to appropriate treatment should be mandatory when dealing with potentially dependent participants, particularly in areas where formal emergency care units or rehabilitation centers may not be available. Eighth, the important question of how to deal with the previously mentioned high comorbidity rates between alcohol-related disorders and depressive, anxiety, and trauma-related disorders was bypassed in many studies in this review, either by excluding individuals exhibiting symptoms of such disorders or by not assessing other psychopathology besides AUD at all. Apart from Sorsdahl et al. (98), who found that their alcohol intervention also reduced depressive symptoms, none of the remaining studies controlled for comorbidities within their analyses. Being aware of such symptomatology is important, as these comorbidities may be associated with poorer treatment responses (149). Ninth and finally, when referring to MI it is important to keep in mind that only some studies employ it as it has initially been proposed and elaborated by Miller and Rollnick (117). Most of the RCTs in this review use elements of MI or MI-based interventions such as the WHO ASSIST-linked brief intervention for hazardous and harmful substance use (81) which are adapted versions of the original format.

### Limitations of the Present Systematic Review

The present systematic review has several limitations. First, it is important to note some methodological factors when interpreting the reported effect sizes. All four high between-condition effect sizes might have been inflated since they were calculated from completer-only and not imputed values with the study by Sorsdahl et al. (98) yielding considerable attrition (38% and 58% in the treatment groups and 40% in the control group). Further, the study by Omeje et al. (87) only included completers of all 20 therapy sessions into their analyses while conducting only one very short-term FU assessment (two weeks post-intervention). Additionally, as this review gives preference to standardized assessment scales and/ or standard drinks per day as outcomes, where available we used these measures for effect size calculations. Only for four studies we had to use other variables depicting alcohol consumption (87, 88, 95, 100). For these studies it has to be kept in mind that the outcomes are less commonly-utilized scales lacking comprehensive validation studies and thus results might be less reliable. Furthermore, Hedge’s *g* does not take pretest differences into account. As the majority of authors reported no differences between groups at baseline, we do not assume a strong bias occurred in this regard. Second, the heterogeneity of the included studies precluded the pooling of outcomes in a meta-analytic manner. Instead, we based our analysis of evidence on the calculation and descriptive comparison of unpooled effect size values. Third, our literature search was limited to studies published in the English language. Apart from a potential selection bias due to language, this additionally entails the risk of publication bias, as significant results are more likely to be published in English-language journals (150). Fourth, due to our selection of search terms, the word ,alcohol’ had to be included in the title, abstract or key words of articles and therefore it cannot be completely ruled out that we failed to identify intervention studies in LMIC which, for example, used alcohol consumption as a secondary outcome. Also, though unlikely, we may have missed records as we did not search for each LMIC included in the World Bank country classification list separately. Finally, we only included randomized-controlled trials which can be seen as a strength of this review, but also entails the risk of disregarding findings originating from other designs such quasi-experimental studies.

## Conclusions

This systematic review further supports the emerging evidence base demonstrating that the use of pragmatic psychosocial interventions can effectively reduce hazardous and harmful alcohol use in low-resource settings. Apart from the complete dearth of intervention research in LMIC-regions affected by conflict, the research gap is increasingly being addressed, with many studies presenting pioneering and promising work on this endeavor.

In summary, the following aspects can be derived from existing research: (1) multiple contact interventions seem to work better than single contact interventions and (2) interventions with sessions of 45 min or longer were on average more effective than those with shorter sessions. However, overall individual counseling intensity across all sessions was still short in virtually all studies (≤150 min). Also, (3) group interventions do not seem to be less effective when compared to individual interventions. (4) The finding that just over 50% of those studies using MI-based approaches yielded a notable treatment effect compared to control entails the necessity of considering alternatives to MI-based approaches which should be tested in future trials. (5) The additional value of therapeutic components applied to enhance MI-based interventions, such as cognitive techniques, remains inconclusive and (6) the finding that, often, assessment or even mere monitoring of alcohol-related symptoms may already reduce drinking cannot be ignored. Finally, (7) in most cases interventions were conducted by trained paraprofessionals suggesting this as a feasible concept in LMIC. However, evaluations of the delivery model itself, including details on an adequate intensity of training and supervision of lay counselors, are lacking.

The RCTs of the current review have provided us with a valuable knowledge base, however, there is room for the improvement of service provision and accompanying research in LMIC settings. We therefore would like to encourage the generation of more research in order to enhance and strengthen the implementation of evidence-based, sustainable and accessible interventions targeting alcohol abuse in LMIC settings.

## Data Availability Statement

The original contributions presented in the study are included in the article/**Supplementary Material**; further inquiries can be directed to the corresponding author.

## Author Contributions

MP, VE, and FN contributed to the conception and design of the review. MP and VE conducted the study selection. MP extracted the data from the selected articles, conducted the data analysis, and drafted the manuscript with supervision from VE. MP and VE contributed to the interpretation of data. All authors contributed to the article and approved the submitted version.

## Conflict of Interest

The authors declare that the research was conducted in the absence of any commercial or financial relationships that could be construed as a potential conflict of interest.
