# Supplementary material for: Effectiveness of Psychosocial Interventions Targeting Hazardous and Harmful Alcohol Use and Alcohol-Related Symptoms in Low- and Middle-Income Countries: A Systematic Review
Source: Front Psychiatry. 2020 Aug 7;11:768. doi: 10.3389/fpsyt.2020.00768 (PMC7438922; doi:10.3389/fpsyt.2020.00768)
Supplement: Supplementary file 2 [file Table_1.pdf]

Supplementary Table 1: Additional study characteristics.

Study	Eligibility criteria (inclusion) alcohol-related inclusion criteria in bold	Exclusion criteria	Supervision for facilitators/ assurance of fidelity		Rationale/ special characteristics and components of intervention for treatment- and control group (common/ established components in bold)	Duration per component / session	Number of sessions
Assanangkorn-chai et al., 2015 (101)	• **ASSIST score 11-26 for alcohol**<br>• age 16–65 years<br>• willing to participate in 6-month follow-up study<br>• resided within a 20-km radius from the hospital and able to provide contact details	• no permanent residence<br>• exhibited aggressive behavior<br>• known to have been involved in serious criminal activity with risk of imprisonment during the subsequent six months<br>• severe cognitive impairment	• supervision: n/r<br>• adherence: checklist of steps taken in the BI and SA procedures was applied during the sessions, all interventions were recorded (project supervisor reviewed first 20 sessions and all checklists, followed by monthly checks on randomly selected records	TG	**BI using the ASSIST-linked BI for hazardous and harmful substance use,** 10-step BI-procedure: Feedback Report Card and Self-Help Strategies Manual used to discuss with the patient the meaning of the score and strategies for reducing or stopping their substance use (focus of intervention was on the substance that resulted in the highest score on the ASSIST or that was of most concern to the participant)	M=8.8 min., range 5-13 min. (could take up to 15 min.)	single session
				CG	SA: P. received feedback on their ASSIST score and its meaning, they were simply advised to stop or reduce their substance use	M=3.9 min., range 3-6 min.	single session
Babor et al., 1992 (79) [a]	**regular inclusion criteria (for those P. who DO NOT want to quit drinking):**<br>• **males: EITHER A) 29 SD per week OR B) 8 SD in one occasion two or more times per month**<br>• **females: EITHER A) 19 SD per week OR B) 5 SD in one occasion two or more times per month**<br>**reduced inclusion criteria (for those P. who DO want to quit drinking or have tried before):**<br>• **males: EITHER A) 25 SD per week OR B) 8 SD in one occasion once or more times per month**<br>• **females: EITHER A) 17 SD per week OR B) 5 SD in one occasion once or more times per month**<br>• **additional criteria (only invoked if P. was just below the formal inclusion criteria):**	• prior or current treatment for alcoholism, drug abuse, liver disease or mental disorder (only if total abstinence from alcohol was recommended to P. by a physician)<br>• P. received advice to refrain completely from drinking alcohol by doctor or other professional<br>• frequent morning drinking (considered evidence for alcohol dependence)<br>• extremely high amounts per day (≥150 g)<br>• pregnancy<br>• no permanent residence or employment	• no supervision once training completed<br>• assurance of fidelity: n/r, only that site visits included a procedure for reviewing the training of health advisors in the different centres	TG1	BI condition:<br>1. 20 min. health interview (WHO composite interview)<br>2. 5 min. of simple advice (same as CG1)<br>3. 15 min. of counseling introducing a '**systematic problem-solving approach**' as possibility of changing one's drinking habits (introduction of the Problem-Solving Manual with the goals: (1) development of therapeutic relationship and coping with resistance (2) work through first 3 sections together (3) identification of a friend or relative who could help the P.)<br>4. P. received the manual and were encouraged to use it in the development of a "habit-breaking-plan"<br>(some investigators decided to add an optional "extended counseling" condition consisting of three follow-up visits after the brief counseling session to provide the patient with periodic support and encouragement (in Mexico and Russia))	40 min.	single session
				TG2	Simple advice condition (SA):<br>1. 20 min. health interview (WHO composite interview)<br>2. 5 min. of simple advice (emphasis on possible relationship between problems mentioned by P. during health interview and drinking, P. were made to realize that they were in the 'heavy drinking category', concepts of standard drinks and drinking limits were introduced)	25 min.	single session

	**concern about drinking by self or family; accidents in which alcohol intake was involved; tardiness or absence from work because of drinking; alcohol use to relieve stress, anxiety or depression; desire to cut down drinking**<br>• age 18–70 years			CG	20 min. health interview (WHO composite interview)	20 min.	single session
Kalichman et al., 2007 (95)	• **current use of alcohol (AUDIT >0)**	• none specified	• weekly supervision meetings with the project manager and a registered counseling psychologist<br>• flipchart guiding the counselor through the session content	TG	1. information/education component (amongst other components): facts about HIV-transmission and risk behaviors, local prevalence, clarification of HIV myths and misconceptions<br>2. **motivational interviewing**: feedback on individual alcohol consumption (according to AUDIT) and associated risks, motivational counseling techniques, discussion about alcohol in sexual contexts using P.'s self-identified risk situations<br>3. behavioral self-management and sexual communication skills building exercises: elaboration of alcohol as major trigger for high-risk behaviors, strategies to reduce risks	1. 20 min.<br>2. 20 min.<br>3. 20 min.	single session
				CG	information/education component: same as TG component 1.	20 min.	single session
Kalichman et al., 2008 (96)	• **having drunk alcohol in the previous month**<br>• age ≥18 years	• none specified	• weekly supervision meetings with the project manager<br>• flipchart guiding the counselor through the session content, debriefment of facilitators and review of session guides after each group by field supervisor	TG	1. information/education component (amongst other components): facts about HIV-transmission and risk behaviors, local prevalence, clarification of HIV myths and misconceptions<br>2. **motivational interviewing**: feedback on individual alcohol consumption (according to AUDIT) and associated risks, personalized brochure, motivational counseling techniques, discussion about alcohol in sexual contexts using P.'s self-identified risk situations<br>3. behavioral self-management and sexual communication skills building exercises: elaboration of alcohol as major trigger for high-risk behaviors, strategies to reduce risks	1. 60 min.<br>2. 60 min.<br>3. 60 min.	single session
				CG	information/education component: same as TG component 1.	60 min.	single session

L'Engle et al., 2014 (102)	• AUDIT score 7-19<br>• female<br>• age ≥18 years<br>• self report of being a sex worker<br>• lived in Mombasa and planned to reside there for the next 12 months	• diagnosis of gonorrhea, chlamydia or trichomoniasis at time of enrollment	• monthly supervision/ quality assurance of intervention delivery by an alcohol intervention expert through direct observation of counseling sessions, meeting with the nurse counselors and presentation of cases, and review of data, assessment, and plan notes	TG	**WHO BI for Alcohol Use**: presentation of individual AUDIT-screening results, **motivational interviewing** techniques such as identification and discussion of risks and consequences from drinking, soliciting P.s' commitment to reduce drinking, identifying the goal of reduced drinking or abstinence, developing a habit-breaking plan, discussing high-risk situations and coping strategies, increasing self-efficacy for changing behavior by providing feedback and encouragement for change; cultural adaptations included the development of visuals for less literate P. (e.g. illustrations of physiological consequences of alcohol use and depictions of risky situations relevant to female sex workers, ladder image to assess motivation and readiness to change because the original ruler image was not understood) and suggestions to reduce risky drinking before engaging in sexual activity	20 min.	6 monthly sessions
				CG	Nutrition intervention (based on Kenyan National Guidelines on Nutrition and HIV/ AIDS): assessment of women's nutritional status, nutritional needs for women and their children and/or women living with HIV, development and monitoring of a nutrition care plan	20 min.	6 monthly sessions
Mertens et al., 2014 (97)	• **subsample: ASSIST-SSIS >11-26 for alcohol (StD=12 g of Ethanol)**<br>• (in the original study eligibility criterion was *either* heavy drinking or regular drug use)	• "too ill to participate"<br>• had no phone and therefore could not be followed	• supervision meetings weekly for 6 weeks and monthly thereafter<br>• listening to records of interventions and feedback by trainer, random selection of tapes scored by trainer	TG	BI based on **motivational interviewing** plus a referral resource list for drinking and drug use<br>(not further specified)	10 min.	single session
				CG	referral resource list for drinking and drug use	n/r	n/r
Nadkarni et al., 2017 (89)	• **AUDIT-score 12-19**<br>• Male<br>• age 18-65 years	• in need of emergency medical treatment or inpatient admission<br>• unable to communicate clearly<br>• intoxicated at the time of screening	• group supervision (weekly) and individual supervision (twice monthly) led by a peer lay counselor<br>• assessment of fidelity e.g. using counselor's clinical records, peer and expert ratings of audio recordings	TG	3-phase treatment based on **motivational interviewing**<br>1. detailed assessment and personalized feedback<br>2. helping the patient to develop cognitive and behavioral skills and techniques, consisting of drink refusal skills, handling of peer pressure, problem-solving skills, and handling of difficult emotions<br>3. relapse prevention and management using the skills aquired in the middle phase<br>+EUC (same as CG)	30-45 min. per session (M=42.4 min., range: 40.9-43.7)	1-4 weekly or fort-nightly sessions (M=2.8 sessions, 95% CI 2.7-3.0)

				CG	EUC: consultation with the Primary Health Center physician (usual care), provision of the AUDIT screening and of a contextualised version of the WHO Mental Health Gap Action Programme guidelines for harmful drinking, including when and where to refer patients for specialist care (enhanced)	n/r	n/r
Noknoy et al., 2010 (105)	• **AUDIT score ≥8 (StD=10 g of ethanol)**<br>• age 18–65 years	• clinical diagnosis of alcohol dependence (DSM-IV)<br>• history of any liver disease<br>• regular morning drinking<br>• recent consumption of extremely high amounts per day (men >120 g, women >80 g)<br>• neurological disease and psychiatric disorders<br>• pregnancy	• supervision: n/r<br>• fidelity to motivational interviewing not assessed	TG	**Adapted Motivational Enhancement Therapy (MET):** patient-centered **motivational interviewing** style, starting with an evaluation of the patient's ability to change his drinking habits according to the following stages of change:<br>1. P. in pre-contemplation stage: main technique was feedback, using reflection and questioning techniques to elicit motivation<br>2. P. in contemplation stage (amongst other components): working with ambivalence using pros and cons technique, empathic counseling style and encouragement of the P.'s self-efficacy to support change<br>3. P. in determination stage: options on how to reduce drinking behavior are provided, self-commitment, setting appropriate goals, plan with measurable goals in changing drinking behavior was made for the action phase<br>4. P. in maintenance stage: relapse-prevention procedures	15 min.	3 sessions: on day 1, at 2 weeks and at 6 weeks after baseline assess-ment
				CG	assessment only	n/r	single session
Omeje et al., 2018 (87)	• **severe level of AUD according to AUDS (self-administered; no cut-off specified)**<br>• **alcohol-related irrational beliefs according to AIBS (self-administered; no cut-off specified)**<br>• HIV-positive	• none specified	n/r	TG	Intervention based on **rational emotive cognitive behavior therapy,** including:<br>1. Behavioral techniques: teaching the participants practical techniques to help them to cope with AUD symptoms, such methods for planning and managing their daily schedule and for distracting themselves from unhealthy thoughts about drinking<br>2. Emotive techniques: to help the study participants to change their negative thoughts on an emotional level; humorous methods, alcohol-related poems, and native satiric songs related to alcohol were used to generate feelings, which could help to challenge and change negative thoughts toward alcohol<br>3. Cognitive/behavioral techniques: disputation of alcohol-related irrational thoughts; P. were taught to overcome the discomfort and anxiety caused by irrational beliefs (e.g. wait for the urge to pass by distracting themselves, dispute the irrational beliefs, eliminate the activating event)	50 min.	20 sessions held twice per week for 10 con-secutive weeks

				CG	waitlist	-	-
Pal et al., 2007 (100)	• **AUDIT score 8–24**<br>• male	• significant medical or psychiatric illnesses	n/r	TG	BI based on **motivational interviewing** (amongst other components): feedback regarding the harmful consequences of drinking while emphasizing the P.'s personal responsibility to change and facilitating self-efficacy and optimism, exploring and discussing alternatives to drinking and implementation of "alternative highs" and coping in the subsequent period, evaluation of high-risk situations	45 min.	2 sessions separated by a 3–5-day gap
				CG	Simple Advice (SA): empathic expression of concern based on consequences, with an advice to cut down or stop alcohol use	5 min.	single session
Papas et al., 2011 (106)	• **any alcohol use in the past 30 days**<br>• **AUDIT-C score ≥3 OR ≥6 drinks per occasion at least monthly (StD=14g of ethanol)**<br>• HIV-infected<br>• enrollment as outpatient at Eldoret clinic<br>• ARV-eligible or ARV-initiated in the past 12 months<br>• spoken knowledge of Kiswahili<br>• living within one hour travel distance from clinic	• active psychosis or suicidality<br>• attendance in the past year at an existing alcohol peer support group or participation in the study's group CBT pre-pilot development	• supervision conducted via telephone during latter stages of trial<br>• all CBT group sessions videotaped and monitored weekly by PI<br>• 50% of sessions (n=18 sessions) randomly selected and rated by 2 raters using a fidelity rating system	TG	**Adapted Cognitive-Behavioral Therapy (CBT)**: amongst other components HIV/alcohol education, reasons for drinking and quitting drinking, collective "quit day" after second session, coping with triggers urges and high-risk situations, risky decisions leading to drinking, problem-solving skills, alcohol refusal skills, development a long term plan<br>(positive saliva test for alcohol consumption in the last 1 to 6 h before sessions precluded P. from attending the session due to the possibility of creating alcohol triggers for other P.)	90 min.	6 weekly sessions
				CG	routine medical care provided in the HIV-outpatient clinic	n/r	n/r
Peltzer et al., 2013 (93)	• **AUDIT score ≥8 (men) / ≥7 (women)**<br>• age ≥18 years<br>• new TB patients (anti-TB treatment for less than one month)	• none specified	• counselors were assessed for adherence to the brief counseling protocol<br>• bi-weekly support visits by the project trainers during implementation<br>• monthly visits by research staff for support with technical aspects<br>• quality control: counselors filled in a patient monitoring form for each session	TG	BI guided by the **Information-Motivation-Behavioral Skills (IMB) Model**, consisted of following steps:<br>1. To identify any alcohol-related problems mentioned in interview<br>2. To introduce the sensible drinking leaflet, emphasize the idea of sensible limits, and make sure that patients realize that they are in the high-risk drinking category<br>3. To provide feedback on the relationship between alcohol and TB treatment<br>4. To work through the first 3 sections of the problem solving manual while mentioning the value of reviewing the other sections<br>5. To describe drinking diary cards<br>6. To identify a helper<br>(includes the use of **motivational interviewing** techniques)	15-20 min.	2 sessions: on day 1 and within one month after baseline evaluation
				CG	P. received a health education leaflet on responsible drinking	-	-

Pengpid et al., 2013 (103)	• **AUDIT score 8–19 (men) / 7–19 (women)**<br>• age ≥18 years	• current treatment for alcoholism<br>• mental impairment<br>• pregnancy	• bi-weekly supervision by the project manager<br>• weekly supervision by a trained counselor mentor<br>• in addition, research assistants were able to report to their coordinators regarding problems<br>• research assistants will be observed "in vivo" for adherence to the protocol	TG	**BI guided by the Information-Motivation-Behavioral Skills-Model**, consisted of following steps:<br>1. To identify any alcohol-related problems mentioned in interview<br>2. To introduce the sensible drinking leaflet, emphasize the idea of sensible limits, and make sure that patients realize that they are in the medium-risk drinking category<br>3. To work through the first 3 sections of the problem solving manual while mentioning the value of reviewing the other sections<br>4. To describe drinking diary cards<br>5. To identify a helper<br>(includes the use of **motivational interviewing** techniques)	20 min.	single session
				CG	P. received a health education leaflet on responsible drinking (no feedback on alcohol-screening)	-	-
Pengpid et al., 2015 (104)	• **ASSIST score 11–26 (moderate risk) for alcohol**<br>• ASSIST score 4–26 (moderate risk) for tobacco<br>• age 18–60 years<br>• able to participate in a 3- and 6-month follow-up post-intervention<br>• able to give contact details for 2–3 other people	• frequent (>4 times/month) injection of drugs in the last 3 months<br>• acute intoxication<br>• currently going through withdrawal from alcohol or drugs<br>• current treatment for alcoholism, nicotine or other drug abuse<br>• no permanent residence<br>• pending incarceration within the next 3 months<br>• cognitive impairment or severe behavior problems	• supervision was provided to research counselors (frequency not further specified)<br>• random sample (10%) of audio tape recording of intervention sessions reviewed by study coordinator for fidelity to protocol	TG	BI (integrative for both alcohol and tobacco use):<br>1. brief counseling for alcohol use reduction and tobacco use cessation intervention using the **ASSIST-linked BI for hazardous and harmful substance use**<br>2. **BI for heavy drinking smokers**: amongst other components discussion on relationship between drinking and smoking and on potential effects of alcohol consumption on smoking cessation, emphasis on personal responsibility for choosing to change one's behavior, advice to avoid or minimize drinking during the smoking cessation process, development of a change strategy, encouragement of P.'s self-efficacy	n/r	3 sessions within a period of 3 weeks
				CG1	BI (alcohol use only):<br>brief counseling for alcohol use reduction using the **ASSIST-linked BI for hazardous and harmful substance use**	n/r	3 sessions within a period of 3 weeks
				CG2	BI (tobacco use only):<br>brief counseling on tobacco use cessation using the **ASSIST-linked BI for hazardous and harmful substance use**	n/r	3 sessions within a period of 3 weeks

Rendall-Mkosi et al., 2013 (94)	• **risky drinking (>5 drinks at one occasion in the past 3 months OR >7 drinks in a week) over the past 3 months**<br>• age 18–44 years<br>• vaginal sex in the past 3 months<br>• ineffective or no contraceptive use<br>• resided within a 25-km radius of the main town	• pregnancy<br>• ondergone sterilization or hysterectomy	regular meetings between the MI trainer and lay counselors for quality control	TG	**Motivational Interviewing** with both risky drinking and ineffective contraceptive use as targeted behaviors (priority behavior of concern to the P. guided the intervention): amongst other components building rapport, focus on assessing P.'s readiness to chance and perceived confidence in enacting behavior change, development of a behavior change plan, focus on implementation of the behavior change plan, assessing challenges and problem solving, reinforcement of an after-care plan<br>• educational flipchart depicting alcohol and contraceptive information was used<br>• received an information pamphlet on fetal alcohol syndrome (FAS) prevention and a woman's health handbook	n/r	5 sessions over 2 months
				CG1	Group-based life-skills training intervention: Intervention not completed due to logistic problems and poor adherence to the intervention (received an information pamphlet on FAS prevention and a woman's health handbook but no further intervention)	-	-
				CG2	P. received an information pamphlet on FAS prevention and a woman's health handbook but no further intervention	-	-
Segatto et al., 2011 (88)	• **alcohol consumption within 6 hours prior to ER visit (determined by 4 multiple choice questions)**<br>• age 16–25 years<br>• permanent residents in Uberlândia	• no permanent residence<br>• severe physical condition<br>• psychotic disorders or mentally challenged<br>• evident cognitive damage<br>• imprisonment	n/r	TG	BI based on **motivational interviewing**, including: evaluation and feedback on the assessment results, information and guidance, encouraging reflection, establishing a plan for change + educational brochure (same as EB-group)	45 min.	single session
				CG	Educational brochure (EB)-group: received a brochure consisting of three pages on the risks of alcohol consumption and possible ways to consider reduction (e.g. "have fun without drink, avoid drinking competitions, think about establishing a limit"), was read by the P. and discussed with the facilitator	max. 5 min.	single session

Sheikh et al., 2017 (90)	• **clinical diagnosis of alcohol dependence (DSM-IV)**<br>• age 18–65 years<br>• close contact with one or more relatives who are taking an interest in their wellbeing	• significant psychiatric disorder<br>• cognitive impairment (MMSE score <23)<br>• diagnosis of major depression, psychosis or suicidal ideation<br>• had no phone	n/r	TG	Brief relapse prevention intervention from mhGAP-IG with involvement of a close family member, includes:<br>1. discussion of alcohol related problems<br>2. considering P.'s attitude towards alcohol and the problems it has caused them (based on **motivational interviewing** techniques)<br>3. actively educating and involving friends, relatives and self-help groups in providing alternative activities to drinking and helping patients to attend follow-up appointments (relatives were also told to help P. remain abstinent and bring them back if they see any signs of relapse)<br>+ detoxification with diazepam and vitamin supplementation	20 min.	single session
				CG	detoxification with diazepam and vitamin supplementation, no further intervention	-	-
Shin et al., 2013 (92)	• **clinical diagnosis of alcohol abuse or dependence (based on CIDI-SAM)**<br>• age ≥18 years<br>• TB diagnosis and registered for TB therapy<br>• TB treatment initiation in one of the three study sites (in-patient hospital, polyclinic, day hospital)	• impaired liver function<br>• reported opioid use in the past month or positive urine screen for opioids<br>• pregnancy or breastfeeding<br>• inadequate understanding of the study after undergoing informed consent<br>• severe medical or psychiatric impairment	• ongoing supervision (level of supervisor n/r) during course of the study<br>• fidelity: all sessions were audiotaped, review of 10% of audiotapes by fidelity-assessment team (addiction and MI-specialists)	TG1	BI condition:<br>BI incorporating routine aspects of the medical encounter such as assessment of laboratory analysis and clinical response as a way of providing information and feedback regarding alcohol consumption to the patient using **motivational interviewing** techniques	10-15 min. within the standard 45–60-min. TB appointm.	6 monthly sessions
				TG2	NTX condition:<br>Administration of Naltrexone (NTX) paired with brief behavioral compliance enhancement treatment (BBCET)<br>(BBCET: focused intervention to enhance naltrexone adherence and promote changes in drinking behavior through goal-setting and naltrexone-specific feedback; differs from BI in that it does not use motivational interviewing<br>NTX: was administered orally as a daily single dose of 50 mg for 6 months, starting at the time of study enrollment)	5-10 min. within the standard 45–60-min. TB appoint-ments	6 monthly sessions
				TG3	BI+NTX condition:<br>(BI: same as in TG1; NTX: same as in TG2)	15-25 min. within the standard 45–60-min. TB appmts.	6 monthly sessions
				CG	TAU condition:<br>TAU: standard referral to and management by a narcologist only	standard 45–60-min. TB appmts.	6 monthly sessions

Sorsdahl et al., 2015 (98)	• **ASSIST score ≥11 for alcohol**<br>• age ≥18 years	• severely altered mental status<br>• physically incapable of participating due to severe illness<br>• unable to provide detailed location information	• biweekly supervision and debriefing sessions<br>• fidelity: counselors had a checklist to ensure all aspects of the intervention were provided	TG1	ASSIST-linked BI based on **motivational interviewing** (amongst other components): feedback on results of alcohol-screening, information about consequences of alcohol on physical and mental health, building rapport and developing readiness to change, assessing pros and cons of change and eliciting commitment to change	20 min.	single session
				TG2	ASSIST-Linked BI based on motivational interviewing (same as TG 1) + Problem Solving Therapy (PST; involves teaching a client how to use a step-by-step process to solve life problems)<br>PST:<br>1. identification of problems occurring in the P.'s life<br>2. focus on exploring one or more of these problems while the counselor trained the P. a structured approach to addressing problems<br>3. completing homework was required for each session providing an opportunity for P. to apply the skills they acquired during their sessions	20 min. per MI-session/ 45-60 min. per PST-sesssion	single session MI + 4 weekly sessions of PST
				CG	psychoeducation only (brochure providing information on the effects of substance use, no additional counseling)	-	-
Wandera et al., 2016 (99)	• **AUDIT-C score ≥3**<br>• age ≥18 years<br>• HIV-infected<br>• planning to continue receiving care at the clinic for the next 6 months	• Karnofsky clinical performance score <50 (=Requires considerable assistance and frequent medical care)<br>• pregnancy	• counselors received individual ongoing support<br>• refresher training to emphasize adherence to treatment protocol during study implementation<br>• no formal assessment of treatment fidelity and adherence but continuous implementation support to ensure that the interventions were provided per protocol	TG	1. BI based on **motivational interviewing** (20-30 min.): amongst other components reflective listening to P. reflect their alcohol use and inquiring about plans to reduce on it, elaboration of possible harms of alcohol and prior attempts to cut down and recognition of such, P. set a personal goal regarding reduction of alcohol consumption in the next 6 months<br>2. Standard Positive Prevention counseling (SPP; 10-30 min.): including supporting adherence to medications, encouraging HIV disclosure to sexual partners and information about safer sex practices, preventing sexually transmitted infections, avoiding alcohol and substance use, information about the risks of alcohol use and encouragement to reduce alcohol intake	30-60 min.	single session
				CG	SPP: same as in TG	10-30 min.	single session

Witte et al., 2011 (91)	• **AUDIT score ≥8**<br>• female<br>• age ≥18 years<br>• self report of being a sex worker<br>• having engaged in unprotected sexual intercourse in the past 90 days with a paying sexual partner<br>• currently enrolled in program of the national AIDS foundation	• severe cognitive or psychiatric impairment	• facilitators received routine monitoring and supervisory feedback on a weekly basis by clinical and task supervisors<br>• all intervention sessions were audiotaped and a random sample of 10% for each facilitator was reviewed by research staff<br>• reviews of intervention quality assurance (adherence) using a checklist that identified any deviation of content or time	TG	HIV-SRR+MI condition:<br>HIV-SRR: same as in TG1<br>MI: two sessions of **motivational interviewing** (one session preceding HIV-SRR and one following) with particular emphasis on alcohol use in the context of HIV risk which included:<br>1. education about the benefits of reducing alcohol intake; encouragement to consider their current levels of alcohol use and identify their state of change; pros and cons of reducing their alcohol intake in order to develop ambivalence<br>2. focus on motivation to change with particular emphasis on alcohol use in the context of HIV risk; identification of behaviors for change; skills to increase self-efficacy by using change talk, development of long-term goals	90 min.	4 weekly sessions +2 additional MI-sessions
				CG1	HIV-SSR condition:<br>HIV-SSR: relationship-based intervention (unit of attention = woman and her paying sexual partner; focused on building knowledge and skills such as identifying safety risks, negotiating safer sex, and avoiding unsafe situations; alcohol and drug use was covered as a contributing factor to sexual risk (not as comprehensively as the MI arm; education on how alcohol affects communication skills and sexual behaviors and increases risk for sexual HIV and STI; three group discussions about substances as triggers and how they contribute to risk; overall consequences of drug and alcohol use are not addressed explicitly unless raised as a concern, no motivational strategies)	90 min.	4 weekly sessions
				CG2	wellness promotion condition:<br>focused on relaxation, the importance of exercise, the harm of smoking and healthy diet and nutrition (matched to HIV-SRR in duration, timing and structure)	90 min.	4 weekly sessions

[a] intervention descriptions listed correspond to the study's core design. The six LMIC study centers included in this review applied the following designs: Bulgaria=3 conditions: core design; Costa Rica=3 conditions: no CG (because considered unethical), SA, BI, extended counseling condition (initial brief counseling condition+3 more sessions with the healthworker to monitor progress during the next 6 months); Kenya=3 conditions: core design; Mexico=6 conditions: CG 1 (no intervention), CG 2 (20 min. of general health counseling), SA 1 (SA only), SA 2 (SA+20 min. of general health counseling), BI, extended counseling condition; former USSR=4 conditions: CG, SA, BI, extended counseling condition; Zimbabwe=4 conditions: CG, SA, BI, supplemental counseling condition (initial brief counseling condition+general health counseling that dealt with smoking, diet and exercise).
AIBS: Alcohol-related Irrational Belief Scale, AUDIT: Alcohol Use Disorder Identification Test, AUDS: Alcohol Use Disorder Scale, ASSIST-SSIS: ASSIST-Specific Substance Involvement Scores, MMSE: Mini-Mental State Examination, n/r: not reported, P.: participant, SD: standard drinks.
