# Supplementary material for: Effectiveness of Psychosocial Interventions Targeting Hazardous and Harmful Alcohol Use and Alcohol-Related Symptoms in Low- and Middle-Income Countries: A Systematic Review
Source: Front Psychiatry. 2020 Aug 7;11:768. doi: 10.3389/fpsyt.2020.00768 (PMC7438922; doi:10.3389/fpsyt.2020.00768)
Supplement: Supplementary file 3 [file Table_2.pdf]

Supplementary Table 2: Detailed risk of bias assessment.

Study	**Selection Bias** (sequence generation)	**Selection Bias** (allocation concealment)	**Performance Bias** (blinding of subjects)	**Detection Bias** (blinding of outcome assessment)	**Attrition Bias** (handling of incomplete outcome data)	**Reporting Bias** (selective reporting)
Assanangkorn-chai et al., 2015 (101)	Unclear risk	Low risk	Low risk	High risk	Low risk	Low risk
	block randomization but generation of random sequence not specified	sequentially numbered opaque sealed envelopes used for concealment	P. not blinded to the intervention condition but unlikely to affect outcomes	same interviewers conducted baseline and all FU-interviews; incomplete blinding	• very high drop-out rates in both groups at FU2 (35-40%) but attrition analysis conducted<br>• flow-chart presented<br>• analysis of outcome data: intent-to-treat	trial prospectively registered with ANZCTR; all prespecified outcomes of interest reported
Babor et al., 1992 (79) [a]	Low risk	Low risk	Unclear risk	Unclear risk	High risk	Unclear risk
	gender- and age-stratified block randomization using a lottery system	sealed envelopes used for concealment	not specified whether P. were blind to condition assignment	no blinding procedures described, only that assessors at baseline were different from those at FU in all centers except Bulgaria	• analyses conducted separately by the different centers<br>• none of the centers reports attrition analyses or flow charts<br>• reasons for drop-out are described very roughly if at all<br>• analysis of outcome data: completer	trial not prospectively registered and no trial protocol published
Kalichman et al., 2007 (95)	Low risk	Unclear risk	Low risk	Unclear risk	Low risk	Unclear risk
	randomly generated list of counseling session scheduling slots (individual randomization)	not stated	P. not blinded to the intervention condition but unlikely to affect outcomes	not stated	• high drop-out rates in all groups (<20%) but attrition analysis conducted<br>• flow-chart presented (but no mention of reasons for attrition)<br>• analysis of outcome data: intent-to-treat	trial not prospectively registered and no trial protocol published
Kalichman et al., 2008 (96)	Unclear risk	Unclear risk	Unclear risk	Low risk	Low risk	Unclear risk
	randomization procedure not specified	allocation done by independent person but concealment method not stated	not specified whether P. were blind to condition assignment	FU assessors blinded to treatment allocation	• attrition analysis conducted<br>• flow-chart presented<br>• analysis of outcome data: per protocol (P. who completed one FU minimum)	trial not prospectively registered and no trial protocol published

L’Engle et al., 2014 (102)	Low risk<br>computer-generated block randomization stratified by treatment center	Low risk<br>sequentially numbered opaque sealed envelopes used for concealment	Low risk<br>P. not blinded to the intervention condition but unlikely to affect outcomes	Low risk<br>FU assessors blinded to treatment allocation	Low risk<br>• no attrition analysis but low attrition<br>• flow-chart presented<br>• analysis of outcome data: intent-to-treat	Low risk<br>trial prospectively registered with clinicaltrials.gov; all prespecified outcomes of interest reported
Mertens et al., 2014 (97)	Unclear risk<br>randomization procedure not specified	Low risk<br>sealed envelopes used for concealment	Unclear risk<br>not specified whether P. were blind to condition assignment	Low risk<br>FU assessors blinded to treatment allocation	Low risk<br>• attrition analysis conducted<br>• flow-chart presented<br>• analysis of outcome data: completer but controlling for baseline variables on which the non-FU-attenders differed in order to monitor selective drop-out (results remained consistent)	Unclear risk<br>trial not prospectively registered and no trial protocol published
Nadkarni et al., 2017 (89)	Low risk<br>block randomization list generated by an independent statistician	Low risk<br>sequentially numbered opaque sealed envelopes used for concealment	Unclear risk<br>not specified whether P. were blind to condition assignment	Low risk<br>FU assessors blinded to treatment allocation	Low risk<br>• attrition analysis conducted<br>• flow-chart presented<br>• analysis of outcome data: intent-to-treat	Low risk<br>trial prospectively registered with ISCRTN; all prespecified outcomes of interest reported
Noknoy et al., 2010 (105)	Low risk<br>block randomization using a standard randomization table	Low risk<br>sealed envelopes used for concealment	Low risk<br>P. in control condition received a cover story ("trial focuses on health behavior")	Low risk<br>FU assessors blinded to treatment allocation	Low risk<br>• no attrition analysis but low attrition<br>• flow-chart presented<br>• analysis of outcome data: intent-to-treat	Unclear risk<br>trial not prospectively registered and no trial protocol published
Omeje et al., 2018 (87)	Low risk<br>computer-generated individual randomization	Low risk<br>P. picked envelope containing the condition from a container	Unclear risk<br>not specified whether P. were blind to condition assignment	High risk<br>all instruments were self administered by the P. who were most likely non-blinded, therefore high risk of detection bias	High risk<br>• reported only the data of those P. that attended all 20 group therapy sessions<br>• flow-chart presented (but information that P. who missed 1+ session(s) were excluded from analyses is not apparent from flow-chart; drop-outs are not reported)<br>• analysis of outcome data: completer	Unclear risk<br>trial not prospectively registered and no trial protocol published

Pal et al., 2007 (100)	Low risk<br>coin tossing (individual randomization)	Unclear risk<br>not stated	Unclear risk<br>not specified whether P. were blind to condition assignment	Low risk<br>FU assessors blinded to treatment allocation	Low risk<br>• no attrition analysis or flow-chart but drop-out generally very low (3-4%)<br>• analysis of outcome data: completer	Unclear risk<br>trial not prospectively registered and no trial protocol published
Papas et al., 2011 (106)	Low risk<br>gender-stratified simple block randomization procedure	Low risk<br>P. drew paper with concealed condition from a jar (supervised by staff)	Low risk<br>P. not blinded to the intervention condition but unlikely to affect outcomes	High risk<br>non-blinded FU assessors were responsible for both recruiting and interviewing P.	Low risk<br>• no attrition analysis but low attrition<br>• flow-chart presented<br>• analysis of outcome data: intent-to-treat	Low risk<br>trial prospectively registered with ICTRP; all prespecified outcomes of interest reported
Peltzer et al., 2013 (93)	Low risk<br>"secure remote randomization service" (cluster randomization of public primary health care facilities)	Unclear risk<br>not stated	Low risk<br>P. not blinded to the intervention condition but unlikely to affect outcomes	Low risk<br>FU assessors blinded to treatment allocation	Low risk<br>• very high drop-out rates in all groups (20-60%) but attrition analysis conducted<br>• flow-chart presented<br>• analysis of outcome data: intent-to-treat	Low risk<br>trial prospectively registered with PACTR; all prespecified outcomes of interest reported
Pengpid et al., 2013 (103)	Low risk<br>computer-generated block randomization	Low risk<br>sequentially numbered opaque sealed envelopes used for concealment	Low risk<br>P. not blinded to the intervention condition but unlikely to affect outcomes	Low risk<br>FU assessors blinded to treatment allocation	Low risk<br>• high drop-out in all groups (>20%) but attrition analysis conducted<br>• flow-chart presented<br>• analysis of outcome data: intent-to-treat	Low risk<br>trial prospectively registered with PACTR; all prespecified outcomes of interest reported
Pengpid et al., 2015 (104)	High risk<br>hospital used as unit of randomization	High risk<br>not stated (but high risk connected to randomization method)	Low risk<br>P. not blinded to the intervention condition but unlikely to affect outcomes	Low risk<br>FU assessors blinded to the respective hospital's randomization status	Low risk<br>• high drop-out rates in all groups (>20%) but attrition analysis conducted<br>• flow-chart presented<br>• analysis of outcome data: intent-to-treat	Unclear risk<br>trial not prospectively registered and no trial protocol published

Rendall-Mkosi et al., 2013 (94)	Low risk	Low risk	Unclear risk	High risk	Low risk	High risk
	computer-generated individual randomization	sealed envelopes used for concealment	blinding not specified and randomization of individuals rather than sites and intervention over 2 months so P. in CG may have been influenced by contact with those in TG	FU-assessors different from personnel conducting the intervention but blinding to condition was "difficult"	• high drop-out rates in all groups (>20%), no attrition analysis but comprehensive reasoning for attrition in flow-chart<br>• analysis of outcome data: intent-to-treat and completer	trial not prospectively registered and no trial protocol published; not reported if P. randomized to third arm which was abolished received any intervention
Segatto et al., 2011 (88)	Low risk	Unclear risk	Low risk	Low risk	Low risk	Unclear risk
	lottery system (individual randomization)	randomization performed by emergency room personnel not linked to the clinical trial but concealment method not stated	P. were blinded to the intervention applied	FU assessors blinded to treatment allocation	• attrition analysis conducted<br>• flow-chart presented<br>• analysis of outcome data: completer	trial not prospectively registered and no trial protocol published
Sheikh et al., 2017 (90)	Low risk	Low risk	Unclear risk	High risk	Low risk	Unclear risk
	computer-generated individual randomization	sealed envelopes used for concealment	not specified whether P. were blind to condition assignment	not stated if assessor was blind but the presence of the relative/ co-therapist might have influenced the P.'s answers	• 100% retention at FU after 2 months<br>• flow-chart presented<br>• analysis of outcome data: completer	trial not prospectively registered and no trial protocol published
Shin et al., 2013 (92)	Low risk	High risk	Low risk	Unclear risk	Low risk	Low risk
	computer-generated block randomization stratified by assigned TB provider	no concealment of allocation	P. not blinded to the intervention condition but unlikely to affect outcomes	not stated	• no attrition analysis but low attrition<br>• flow-chart presented<br>• analysis of outcome data: intent-to-treat	trial prospectively registered with clinicaltrials.gov; all prespecified outcomes of interest reported
Sorsdahl et al., 2015 (98)	Low risk	Low risk	Unclear risk	Low risk	Low risk	Low risk
	individual randomization using a random number generator	sequentially numbered sealed envelopes used for concealment	not specified whether P. were blind to condition assignment	FU assessors blinded to treatment allocation	• very high drop-out rates in all groups (40-58%) but attrition analysis conducted<br>• flow-chart presented<br>• analysis of outcome data: intent-to-treat	trial prospectively registered with PACTR; all prespecified outcomes of interest reported

Wandera et al., 2016 (99)	Low risk	Low risk	Unclear risk	Unclear risk	Low risk	Low risk
	computer-generated random numbers for block randomization	sequentially numbered, opaque, sealed envelopes used for concealment	not specified whether P. were blind to condition assignment	same person assessed baseline and FU but not stated if s/he was blind to condition	• drop-out at FU2 very low (2-4%) and attrition analysis conducted<br>• flow-chart presented<br>• analysis of outcome data: intent-to-treat	trial prospectively registered with clinicaltrials.gov; all prespecified outcomes of interest reported
Witte et al., 2011 (91)	Low risk	Unclear risk	Unclear risk	Low risk	Low risk	Unclear risk
	cluster randomization using a random number generator (entities for clustering=women groups; "randomizing by group was more efficient than attempting to accrue enough women in a short enough period of time to then randomize individually to three conditions at one time")	sequence generation centrally done but not stated if allocation was concealed	not specified whether P. were blind to condition assignment	FU assessors blinded to treatment allocation	• high drop-out (33-15% with higher drop-out in TG1 compared to other groups) but attrition analysis conducted<br>• flow-chart presented<br>• analysis of outcome data: intent-to-treat	trial not prospectively registered and no trial protocol published

[a] for the WHO-Multicenter trial (Babor, 1992) the overall report was taken as a basis for assessment of risk of bias. In case deviations were found for specific study centers this was noted here.
FU: follow-up, P.: participant, TG: treatment group.
