# Supplementary material for: Effectiveness of Psychosocial Interventions Targeting Hazardous and Harmful Alcohol Use and Alcohol-Related Symptoms in Low- and Middle-Income Countries: A Systematic Review
Source: Front Psychiatry. 2020 Aug 7;11:768. doi: 10.3389/fpsyt.2020.00768 (PMC7438922; doi:10.3389/fpsyt.2020.00768)
Supplement: Supplementary file 4 [file Table_3.pdf]

Supplementary Table 3: Narrative results reported by the authors of studies excluded from quantitative analyses.

Study	Outcome Variables directly related to alcohol consumption	Assessment Scales [a,b] (alcohol-related outcome/s only)	FU assessment periods (alcohol-related outcome/s only)	(Narrative) Results as reported in studies (alcohol-related outcome/s only)	Retention in TG and CG	Outcome Variables unrelated to alcohol consumption or non-behavioral outcomes
Babor et al., 1992: Bulgaria (Boyadjieva) (151)	(1) average daily alcohol consumption (total amount of alcohol during typical month divided by 30)<br>(2) typical intensity of alcohol consumption per drinking day (total amount of alcohol during typical month divided by the actual no. of drinking days)<br>(3) days drinking during typical month (single item about no. of drinking days)<br>(4) frequency of dependence symptoms (six items, rating scale format)<br>(5) alcohol-related social, legal, medical and employment problems (seven items, dichotomous response format)<br>frequency of expressed concern about the P.'s drinking by close friend or relative (six categories of social relationships to be rated on rating scale format)	(1-6) WHO CIDI	FU: 6 months (post-intervention)	changes in both TGs were n.s. for all outcome-variables suggesting that both simple advice and brief counseling are not effective when applied in a community setting (n=50, all male; female P. did not enter analyses due to too small n)	FU: 76% (across all groups)	• attitudes about alcohol and drunkenness, acceptable forms for heavy drinking and intoxication, reasons for drinking and/or not drinking global depression, sociopathic personality characteristics, typical coping responses to problems, self-confidence

Babor et al., 1992: Costa Rica (Montero) (152)	see 6a	see 6a	see 6a	• sign. reduction in the intensity of drinking (2) across all three groups suggesting no differential effect for the conditions<br>• no changes in typical consumption (1) or drinking days (3) across all three groups<br>• no improvement in dependence scores (4), problem scores (5) or concern scores (6)	FU: 100% (across all groups)	see 6a
Babor et al., 1992: Kenya (Acuda) (153)	see 6a	see 6a	see 6a	• sign. reduction in all measures (1,2,3,4,6) except the problem score (5) in both TGs and the CG<br>• some suggestion that the intensity of drinking (2) changed more in the TGs than in the CG but no clear evidence that the intervention was responsible for the change in alcohol consumption (only a trend, interaction n.s.)	FU: 32% (across all groups)	see 6a
Babor et al., 1992: Mexico (Campillo et al.) (154)	see 6a	see 6a	see 6a	• sign. reduction in all measures (1,2,3,4,5,6) in both the TGs and the CGs so these changes cannot be attributed to the effects of the intervention (no time by condition interaction except for outcome (3))<br>• some evidence (sign. interaction) that the no. of drinking days (3) changed more in the TGs (four days a month) than the CGs (three days a month)	FU: 74% (across all groups)	see 6a

Babor et al., 1992: former USSR (Ivanets & Lukomskaya) (155)	see 6a	see 6a	see 6a	• sign. reduction in all drinking measures except for the problem score and the dependence score (1,2,3,6) in both TGs and CGs, so these changes cannot be attributed to the effects of the intervention<br>• P. in the TGs (all three combined) showed more change than those in the CG (sign. interaction) for typical consumption (1) and intensity of drinking (2)<br>• strong opposition to active monitoring of drinking habits with only few P. returning for the sessions	FU: 71% (across all groups)	see 6a
Babor et al., 1992: Zimbabwe (156)	see 6a	see 6a	see 6a	• sign. reduction in all measures except the problem score (1,2,3,4,6) in both TGs and the CG<br>• differential effects (time by condition interactions) for the combined intervention conditions for typical consumption (1), intensity (2) and no. of drinking days (3)<br>• reductions were uniform across all three types of interventions suggesting that brief counseling did not add to the effect of the simple advice	FU: 92% (across all groups)	see 6a
Kalichman et al., 2008 (96)	(1) drinking in sexual contexts in previous month	*single item about no. of times they drank alcohol before sex*	FU1: 3 months<br>FU2: 6 months (post-intervention)	• intervention effects moderated by alcohol use: lighter drinkers demonstrated sign. more intervention gains than heavier drinkers in the risk-reduction outcome (1); effects occurred at FU1 and dissipated by FU2	FU1: 81% (TG), 85% (CG)<br>FU2: 90% (TG), 89% (CG)	• meeting sex-partners at shebeens; sexual risk and protective behaviors in the previous month<br>• risk-reduction self-efficacy; alcohol outcome expectancies; risk-reduction behavioral intentions; HIV-prevention knowledge; AIDS-related stigmas

Rendall-Mkosi et al., 2013 (94)	(1) being at risk of an alcohol exposed pregnancy (AEP)<br>(2) "risky drinking" (Alcohol-related symptoms in the past year)	*(1) items asking about engagement in risky drinking and no or ineffective use of contraception*<br>(2) AUDIT, CAGE	FU1: 3 months<br>FU2: 12 months (post-intervention)	• declines sign. for both groups in the proportion of P. who met criteria for risky drinking (2), no interaction effect<br>• sign. difference in the decline in the proportion of women at risk for an AEP (1) in the TG at FU1 and FU2<br>• BUT: the reduction in risk for AEP was due mainly to the improved use of contraceptives rather than a reduction in risky alcohol use	FU1: 70% (TG), 74% (CG)<br>FU2: 74% (TG), 77% (CG)	
Shin et al., 2013 (92)	(1) mean no. of abstinent days in the last month of the study<br>(2) mean no. of heavy drinking days (≥4 drinks/ ≥5 drinks per day for women/ men) in the last month of the study	(1,2) TLFB	FU1: 3 months<br>FU2: 6 months (post-enrollment)	• alcohol abstinence days (1) did not differ between naltrexone and non-naltrexone or BCI and non-BCI groups<br>• change in number of heavy drinking days (2) also did not differ among arms.<br>• among P. with a prior alcohol quitting attempt NTX use was associated with more favorable TB outcomes but no difference in terms of abstinent or heavy drinking days<br>• not stated whether or not sign. effects of time have been found	FU1: n/r<br>FU2: 96% (TG1), 94% (TG2), 87% (TG3), 96% (CG)	• favorable TB outcome (cured or completed treatment)<br>• TB adherence (percentage of doses taken as prescribed)

[a] unstandardized/ self-developed scales in *italics.*

[b] reference periods (unless otherwise stated): AUDIT=12 months; WHO CIDI=6 months.

AUDIT: Alcohol Use Disorder Identification Test, CG: control group, FU: follow-up, no.: number, n.s.: non-significant, n/r: not reported, P.: participant, sign.: significant/ significantly, TG: treatment group, TLFB: Timeline Followback, WHO CIDI/CIDI SAM: The World Health Organization World Mental Health Composite International Diagnostic Interview, Substance Abuse Module.
